# Analyzing the genes related to Alzheimer’s disease via a network and pathway-based approach

**DOI:** 10.1186/s13195-017-0252-z

**Published:** 2017-04-27

**Authors:** Yan-Shi Hu, Juncai Xin, Ying Hu, Lei Zhang, Ju Wang

**Affiliations:** 1 School of Biomedical Engineering, Tianjin Medical University, Tianjin, 300070 China; 2 School of Computer Science and Technology, Tianjin University, Tianjin, 300072 China

**Keywords:** Alzheimer’s disease, Functional enrichment analysis, Network analysis, Pathway crosstalk

## Abstract

**Background:**

Our understanding of the molecular mechanisms underlying Alzheimer’s disease (AD) remains incomplete. Previous studies have revealed that genetic factors provide a significant contribution to the pathogenesis and development of AD. In the past years, numerous genes implicated in this disease have been identified via genetic association studies on candidate genes or at the genome-wide level. However, in many cases, the roles of these genes and their interactions in AD are still unclear. A comprehensive and systematic analysis focusing on the biological function and interactions of these genes in the context of AD will therefore provide valuable insights to understand the molecular features of the disease.

**Method:**

In this study, we collected genes potentially associated with AD by screening publications on genetic association studies deposited in PubMed. The major biological themes linked with these genes were then revealed by function and biochemical pathway enrichment analysis, and the relation between the pathways was explored by pathway crosstalk analysis. Furthermore, the network features of these AD-related genes were analyzed in the context of human interactome and an AD-specific network was inferred using the Steiner minimal tree algorithm.

**Results:**

We compiled 430 human genes reported to be associated with AD from 823 publications. Biological theme analysis indicated that the biological processes and biochemical pathways related to neurodevelopment, metabolism, cell growth and/or survival, and immunology were enriched in these genes. Pathway crosstalk analysis then revealed that the significantly enriched pathways could be grouped into three interlinked modules—neuronal and metabolic module, cell growth/survival and neuroendocrine pathway module, and immune response-related module—indicating an AD-specific immune-endocrine-neuronal regulatory network. Furthermore, an AD-specific protein network was inferred and novel genes potentially associated with AD were identified.

**Conclusion:**

By means of network and pathway-based methodology, we explored the pathogenetic mechanism underlying AD at a systems biology level. Results from our work could provide valuable clues for understanding the molecular mechanism underlying AD. In addition, the framework proposed in this study could be used to investigate the pathological molecular network and genes relevant to other complex diseases or phenotypes.

**Electronic supplementary material:**

The online version of this article (doi:10.1186/s13195-017-0252-z) contains supplementary material, which is available to authorized users.

## Background

Alzheimer’s disease (AD) is the most prevalent neurodegenerative disorder and accounts for the majority of people diagnosed with dementia [1]. As a complex and chronic neurological disease, AD affects about 6% of people aged 65 years and older [2], and is responsible for about 480,000 deaths per year around the world [3]. In addition to its affect on the life quality of those suffering from the disorder and their families, AD also causes a severe burden on society. In the USA alone, the health-care costs related to AD are about $172 billion per year [4].

AD can be diagnosed by symptoms such as short-term memory loss, mood swings, learning impairments, and disruptions in daily activities [5]. However, as an age-related and progressive disease, some pathological features of AD (e.g., amyloid deposition, accumulation of neurofibrillary tangles, as well as function and structure changes of brain regions involved in memory) often appear many years prior to clinical manifestations [6, 7]. These pathological changes eventually lead to the damage and death of specific neurons, resulting in the emergence of clinical symptoms.

The cause of AD is still poorly understood although much effort has been dedicated to exploring the pathological and molecular mechanisms of AD via various approaches—e.g., animal models, gene expression profiling, genome-wide association studies (GWAS), neuroimaging techniques, or a systems biology framework [2, 8–11]. It is agreed that AD develops as a result of the combination of multiple factors, including genetic factors, a history of head injuries, depression, or hypertension. Among these factors, it is estimated that about 70% of the risk for AD is attributable to genetics [1, 12]. Established genetic causes of AD include the dominant mutations of genes encoding amyloid precursor protein (*APP*), presenilin 1 (*PSEN1*), and presenilin 1 (*PSEN2*). However, these genes are only responsible for the pathogenesis of AD in about 5% of patients with clinical symptoms appearing in midlife. On the other hand, genetic analyses have suggested that, in complex disorders like AD, individual differences can be caused by many genes and their variants. Genes with various biological functions may act in coordination to increase the risk of AD, with a moderate or small effect exerted by each gene [1]. Consistent with this view, more and more genes—e.g., apolipoprotein E (*APOE*), glycogen synthase kinase 3 beta (*GSK3B*), dual specificity tyrosine-phosphorylation-regulated kinase 1A (*DYRK1A*), and Tau—have been found to be potentially associated with AD [1, 13]. For these genes, although a few plausible candidate genes have been partially replicated, some are considered problematic. This is especially true as high-throughput methods like GWAS are being more widely applied to genetic studies of AD. Under such circumstances, a comprehensive analysis of potential causal genes of AD within a pathway and/or a network framework may not only provide us with important insights beyond the conventional single-gene analyses, but also offer consolidated validation for the individual candidate gene.

In the current study, we implemented a comprehensive curation of AD-related genes from genetic association studies. We then conducted biological enrichment analyses to detect the significant functional themes within these genetic factors and analyzed the interactions among the enriched biochemical pathways by pathway crosstalk analysis. Furthermore, an AD-specific protein network was inferred and evaluated with the human protein–protein interaction network as the background. This study should offer valuable hints for understanding the molecular mechanisms of AD from a perspective of systems biology.

## Methods

### Identification of AD-related genes

The genes genetically associated with AD were collected by retrieving the human genetic association studies deposited in PubMed (http://www.ncbi.nlm.nih.gov/pubmed/). We retrieved publications associated with AD with the searching term ‘(Alzheimer’s Disease [MeSH]) AND (Polymorphism [MeSH] OR Genotype [MeSH] OR Alleles [MeSH]) NOT (Neoplasms [MeSH])’. By July 7, 2015, a total of 5298 reports were retrieved. After reviewing all abstracts of these publications, only the genetic association studies on AD were selected. From the obtained publication pool, we then concentrated on those studies reporting a significant association of gene(s) with AD. In order to reduce the number of potential false-positive genes, the studies reporting insignificant or negative associations were excluded even though some genes in these studies might actually be truly associated with AD. We then reviewed the full reports of each selected publication to make sure that the conclusion was consistent with its contents. In several studies, some genes were found to function cooperatively to exert significant influences on AD, with each gene having a small or mild impact; these genes were also included in our list. In addition, the genes from several GWAS analyses on AD, showing genetic association at a genome-wide significance level, were also included.

### Functional enrichment analysis of genes related to AD

WebGestalt [14] and ToppGene [15] were utilized to detect the biological themes of the AD-related genes. As a web-based bioinformation-mining platform, WebGestalt integrates information from multiple resources to determine the biological themes, including identifying the overrepresented Gene Ontology (GO) terms, amid the candidate gene listing. In this study, only the GO biological process terms with false discovery rate (FDR) value smaller than 0.05 were kept as the significantly enriched ones. ToppGene was used to identify and analyze the enriched biological pathways in the input genes. Pathways with FDR < 0.05 were considered to be significantly enriched.

### Analysis of crosstalks among pathways

We further built crosstalks among pathways to investigate interlinks and interactions of the enriched pathways. To measure the overlap between two pathways, the overlap coefficient (OC) and the Jaccard coefficient (JC) were calculated using the corresponding formulas:

$$ \mathrm{O}\mathrm{C}=\frac{\left| A\cap B\right|}{ \min \left(\left| A\right|,\left| B\right|\right)} $$

and

$$ \mathrm{J}\mathrm{C}=\left|\frac{A\cap B}{A\cup B}\right|, $$

in which A and B are the lists of genes of the two examined pathways. Briefly, the following procedure was adopted to construct the pathway crosstalks:

1. Only pathways with FDR < 0.05 were kept for crosstalk analysis. Meanwhile, pathways with five or fewer candidate genes were discarded because pathways with too few candidate genes might present few or biased connections with other pathways.
2. Counting the common candidate genes of each pathway pair—those pathway pairs with less than two overlapped genes were removed.
3. Measuring the overlap in every pathway pair by the corresponding JC and OC values.
4. Constructing the pathway crosstalk with Cytoscape software [16].

### Compilation of the human protein–protein interaction network

To explore the correlation and interaction among the AD-related genes, we compiled a comprehensive protein–protein interaction (PPI) network, based on which the protein network topological properties of the gene set related to AD were calculated and analyzed. Briefly, the human protein–protein interaction data were obtained from the Protein Interaction Network Analysis (PINA) database (latest release version: May 21, 2014) [17] by pooling and curating the unique physical interaction information from six main public protein interaction databases: BioGRID, IntAct, DIP, MINT, MIPS/MPact, and HPRD. In the meantime, another interactome for *Homo sapiens* [18] that contained 141,296 edges (physical protein interactions) among 13,460 nodes (proteins), consisting of metabolic pathway-related interactions, regulatory and protein–protein interactions, and interaction pairs for kinase and specific substrate, was selected as an additional source of interactome data. After merging the two interactome data by excluding the self-interacting and redundant pairs, the proteins in the list were mapped onto Entrez protein-coding genes for *Homo sapiens* via the Uniprot ID mapping tool (http://www.uniprot.org/uploadlists). Finally, we compiled a relatively comprehensive human physical interactome, which comprised 16,022 genes/proteins and 228,122 interactions (see Additional file 1).

### Construction of the AD-specific protein subnetwork

A subnetwork specific to a given disease can provide us with hints for how the disease-related molecules interact with each other. A network parsimony principle has been demonstrated in the context of biological processes [19]; that is, the molecular networks/pathways often follow the shortest molecular paths between known disease-associated components (disease-related genes or proteins in our case). The Steiner minimal tree algorithm coincides with this biological principle, which uses a greedy heuristic strategy to iteratively link the smaller trees to larger ones until there is only one tree connecting all seed nodes [20]. GenRev [21] was utilized to identify the pathological subnetwork from the human interactome using the curated AD-related genes as input. To assess the non-randomness of the constructed network, 1000 random networks with the same number of vertices and interactions as the AD-specific network were generated using the Erdos-Renyi model in R igraph package [22].

## Results

### Compilation of genes associated with AD

Genes associated with AD were compiled through searching the published genetic association studies on AD in PubMed. Only the publications reporting gene(s) significantly associated with the disease were pooled, and those reporting a negative or insignificant association were excluded. Altogether, from 823 reports, we collected 430 genes reported to be associated with AD (Additional file 2: Table S1; the gene list is referred to as Alzgset). Among them were seven apolipoprotein genes (*APOA1*, *APOA4*, *APOC1*, *APOC2*, *APOC4*, *APOD*, and *APOE*), five genes encoding subunits of nicotinic acetylcholine receptors (*CHRNA3*, *CHRNA4*, *CHRNA7*, *CHRNB2*, and *CHRFAM7A*), four adrenoceptors (*ADRA2B*, *ADRB1*, *ADRB2*, and *ADRB3*), two serotonin receptors (*HTR2A* and *HTR6*), three dopamine degradation genes (*COMT*, *DBH*, and *MAOA*), and one dopamine receptor (*DRD4*). A few transport-related genes were also collected, such as ATP-binding cassette transporters (*ABCA1*, *ABCA2*, *ABCA7*, *ABCC2*, *ABCG1*, and *ABCG2*), a dopamine transporter (*SLC6A3*), a serotonin transporter (*SLC6A4*), two glucose transporters (*SLC2A9* and *SLC2A14*), a folate transporter (*SLC19A1*), and ion transporters (*SLC24A4*). The other genes were those involved in the biological processes related to nitric oxide synthesis (*NOS1* and *NOS3*), immune response (e.g., *IL1A*, *IL6*, *IL10*, and *NLRC3*), as well as mitochondria-specific function (e.g., *MT-ATP6*, *MT-CO1*, *MT-CYB*, and *MTHFR*). Clearly, the genes significantly associated with AD were diverse in function, consistent with the complexity of this mental disorder.

### Biological function enrichment analysis of Alzgset

Functional enrichment analysis revealed a more detailed biological function spectrum of these AD-related genes (see Additional file 2: Table S2). Among the GO terms overrepresented in Alzgset, those related to lipid and/or lipoprotein-related processes, drug reactions, neural development, or synaptic transmission were included. GO terms associated with drug reactions (e.g., response to ethanol, response to nicotine, and response to cocaine) and metabolic processes (e.g., xenobiotic metabolic process) were overrepresented. These results were in line with previous findings that complicated correlations existed between the pathophysiological state of AD and drug abuse [23, 24]. Of significance, top-ranked terms included some lipid/lipoprotein-related processes, including phospholipid efflux, reverse cholesterol transport, cholesterol homeostasis, and lipoprotein metabolic processes. Biological process terms related to synaptic transmission (e.g., positive regulation of transmission of nerve impulse; synaptic transmission, cholinergic; regulation of synaptic transmission, dopaminergic; and regulation of neurotransmitter secretion), dopamine metabolism (dopamine metabolic process), and other neural functions (e.g., synaptic vesicle transport, regulation of neuronal synaptic plasticity, neuron migration, and memory) were also enriched. Meanwhile, GO terms related to immunological function (e.g., T-helper 1 type immune response, positive regulation of interleukin-6 production, and chronic inflammatory response) were overrepresented. The diversity in the function of AD-related genes demonstrated the complexity of the disease.

### Biochemical pathway enriched in Alzgset

Detecting the biological pathways overrepresented among Alzgset may provide useful information about the pathogenic molecular mechanism underlying AD. For Alzgset, 68 enriched pathways were identified (Table 1). Among them, several pathways related to immune processes were included (e.g., cytokines and inflammatory response, cytokine network, dendritic cells in regulating TH1 and TH2 development, and IL-5 signaling), consistent with previous studies [25, 26]. Also, neurotransmitter signaling-related pathways were identified, such as cholinergic synapse, dopaminergic synapse, serotonergic synapse, and so forth. Additionally, in the Alzgset enriched pathway list, there were some pathways related to cell growth and/or survival, including neurotrophin signaling, PI3K-Akt signaling, mTOR signaling, Notch signaling, and so forth, which are vital for cell growth/survival state of neurons in the process of AD [27, 28]. Moreover, metabolism-related pathways, consisting of drug metabolism (cytochrome P450), glutathione metabolism, and metabolism of xenobiotics by cytochrome P450, were also significantly enriched, indicating that related metabolism processes were involved in the etiology and development processes of AD. What is more, the pathway of the intestinal immune network for IgA production was enriched, which might suggest a connection between AD and the intestinal microbiota [29, 30]. Furthermore, pathways involved in osteoclast differentiation and adipocytokine signaling were also detected, complying with prior studies [31–33].

**Table 1:** Pathways enriched in Alzgset^a^

Pathway	*p* value^b^	*p*_BH_ value^c^	Genes included in the pathway^d^
Cytokines and inflammatory response	1.03 × 10^–9^	8.79 × 10^–8^	*CXCL8*, *HLA-DRA*, *HLA-DRB1*, *IL10*, *IL12A*, *IL12B*, *IL1A*, *IL4*, *IL6*, *TGFB1*, *TNF*
cytokine network	9.89 × 10^–9^	3.84 × 10^–7^	*CXCL8*, *IL10*, *IL12A*, *IL12B*, *IL18*, *IL1A*, *IL4*, *IL6*, *TNF*
Hematopoietic cell lineage	1.92 × 10^–7^	5.46 × 10^–6^	*CD14*, *CD33*, *CD36*, *CD44*, *CR1*, *HLA-DRA*, *HLA-DRB1*, *HLA-DRB5*, *IL1A*, *IL1B*, *IL4*, *IL6*, *IL6R*, *MME*, *TNF*
Dendritic cells in regulating TH1 and TH2 Development	3.11 × 10^–7^	8.29 × 10^–6^	*CD33*, *IL10*, *IL12A*, *IL12B*, *IL4*, *TLR2*, *TLR4*, *TLR9*
Ovarian steroidogenesis	5.88 × 10^–6^	1.09 × 10^–4^	*ALOX5*, *CYP19A1*, *FSHR*, *IGF1*, *INS*, *LDLR*, *LHCGR*, *PLA2G4A*, *PTGS2*, *STAR*
IL-5 signaling pathway	9.00 × 10^–6^	1.60 × 10^–4^	*HLA-DRA*, *HLA-DRB1*, *IL1B*, *IL4*, *IL6*
Neurotrophin signaling pathway	1.08 × 10^–5^	1.77 × 10^–4^	*BDNF*, *CAMK2D*, *GSK3B*, *IRS1*, *NGF*, *NGFR*, *NTF3*, *NTRK1*, *NTRK2*, *PIK3R1*, *PSEN1*, *PSEN2*, *SOS2*, *TP53*, *TP73*
HIF-1 signaling pathway	1.12 × 10^–5^	1.77 × 10^–4^	*CAMK2D*, *EIF4EBP1*, *GAPDH*, *HMOX1*, *IGF1*, *IL6*, *IL6R*, *INS*, *NOS3*, *PIK3R1*, *RPS6KB2*, *TF*, *TLR4*, *VEGFA*
NOD-like receptor signaling pathway	1.66 × 10^–5^	2.37 × 10^–4^	*CARD8*, *CCL2*, *CXCL8*, *IL18*, *IL1B*, *IL6*, *MEFV*, *NLRP1*, *NLRP3*, *TNF*
Mechanism of gene regulation by peroxisome proliferators via PPARα	1.95 × 10^–5^	2.69 × 10^–4^	*APOA1*, *CD36*, *INS*, *LPL*, *PIK3R1*, *PPARA*, *PTGS2*, *RXRA*, *SP1*, *TNF*
Th1/Th2 differentiation	2.54 × 10^–5^	3.19 × 10^–4^	*HLA-DRA*, *HLA-DRB1*, *IL12A*, *IL12B*, *IL18*, *IL4*
Antigen-dependent B-cell activation	2.68 × 10^–5^	3.26 × 10^–4^	*FAS*, *HLA-DRA*, *HLA-DRB1*, *IL10*, *IL4*
Oxidative phosphorylation	3.74 × 10^–5^	4.39 × 10^–4^	*COX10*, *COX15*, *MT-ATP6*, *MT-ATP8*, *MT-CO1*, *MT-CO2*, *MT-CO3*, *MT-CYB*, *MT-ND1*, *MT-ND2*, *MT-ND3*, *MT-ND4*, *MT-ND4L*, *MT-ND5*, *MT-ND6*
PI3K-Akt signaling pathway	3.80 × 10^–5^	4.39 × 10^–4^	*COL11A1*, *EFNA5*, *EIF4EBP1*, *FGF1*, *GNB3*, *GSK3B*, *IGF1*, *IL4*, *IL6*, *IL6R*, *INS*, *IRS1*, *NGF*, *NGFR*, *NOS3*, *PCK1*, *PIK3R1*, *PPP2R2B*, *RELN*, *RPS6KB2*, *RXRA*, *SOS2*, *TLR2*, *TLR4*, *TP53*, *VEGFA*, *YWHAQ*
NF-κB signaling pathway	4.83 × 10^–5^	5.42 × 10^–4^	*CD14*, *CXCL8*, *ICAM1*, *IL1B*, *LCK*, *PARP1*, *PLAU*, *PTGS2*, *TLR4*, *TNF*, *TRAF2*, *UBE2I*
Phagosome	7.77 × 10^–5^	8.29 × 10^–4^	*CD14*, *CD36*, *CTSS*, *HLA-A*, *HLA-DQB1*, *HLA-DRA*, *HLA-DRB1*, *HLA-DRB5*, *MBL2*, *MPO*, *NOS1*, *OLR1*, *RAB7A*, *TAP2*, *TLR2*, *TLR4*
Erythrocyte differentiation pathway	9.33 × 10^–5^	9.49 × 10^–4^	*CCL3*, *IGF1*, *IL1A*, *IL6*, *TGFB1*
IL-10 anti-inflammatory signaling pathway	1.82 × 10^–4^	1.69 × 10^–3^	*HMOX1*, *IL10*, *IL1A*, *IL6*, *TNF*
Cells and molecules involved in local acute inflammatory response	1.82 × 10^–4^	1.69 × 10^–3^	*CXCL8*, *ICAM1*, *IL1A*, *IL6*, *TNF*
Toll-like receptor signaling pathway	2.15 × 10^–4^	1.95 × 10^–3^	*CCL3*, *CD14*, *CXCL8*, *IL12A*, *IL12B*, *IL1B*, *IL6*, *PIK3R1*, *TLR2*, *TLR4*, *TLR9*, *TNF*
Free radical induced apoptosis	2.22 × 10^–4^	1.97 × 10^–3^	*CXCL8*, *GPX1*, *SOD1*, *TNF*
Intestinal immune network for IgA production	2.65 × 10^–4^	2.26 × 10^–3^	*HLA-DQB1*, *HLA-DRA*, *HLA-DRB1*, *HLA-DRB5*, *IL10*, *IL4*, *IL6*, *TGFB1*
Selective expression of chemokine receptors during T-cell polarization	3.35 × 10^–4^	2.68 × 10^–3^	*CCL3*, *CCR2*, *IL12A*, *IL12B*, *IL4*, *TGFB1*
B lymphocyte cell surface molecules	3.39 × 10^–4^	2.68 × 10^–3^	*CR1*, *HLA-DRA*, *HLA-DRB1*, *ICAM1*
Phosphorylation of MEK1 by cdk5/p35 downregulates the MAP kinase pathway	3.39 × 10^–4^	2.68 × 10^–3^	*CDK5*, *CDK5R1*, *NGF*, *NGFR*
Complement and coagulation cascades	4.61 × 10^–4^	3.58 × 10^–3^	*A2M*, *C4A*, *C4B*, *CFH*, *CR1*, *F13A1*, *MBL2*, *PLAU*, *SERPINA1*
ABC transporters	5.87 × 10^–4^	4.32 × 10^–3^	*ABCA1*, *ABCA2*, *ABCA7*, *ABCC2*, *ABCG1*, *ABCG2*, *TAP2*
Signal transduction through IL-1R	6.97 × 10^–4^	5.05 × 10^–3^	*IL1A*, *IL1B*, *IL1RN*, *IL6*, *TGFB1*, *TNF*
mTOR signaling pathway	8.19 × 10^–4^	5.83 × 10^–3^	*EIF4EBP1*, *IGF1*, *INS*, *IRS1*, *PIK3R1*, *RPS6KB2*, *TNF*, *VEGFA*
Adhesion and diapedesis of granulocytes	9.49 × 10^–4^	6.65 × 10^–3^	*CXCL8*, *ICAM1*, *IL1A*, *TNF*
TNF signaling pathway	1.12 × 10^–3^	7.69 × 10^–3^	*CCL2*, *FAS*, *ICAM1*, *IL1B*, *IL6*, *MAGI2*, *MMP3*, *PIK3R1*, *PTGS2*, *TNF*, *TRAF2*
MAPK signaling pathway	1.13 × 10^–3^	7.69 × 10^–3^	*BDNF*, *CD14*, *FAS*, *FGF1*, *IL1A*, *IL1B*, *MAPK8IP1*, *MAPT*, *MEF2C*, *NGF*, *NTF3*, *NTRK1*, *NTRK2*, *PLA2G4A*, *SOS2*, *TGFB1*, *TNF*, *TP53*, *TRAF2*
The IGF-1 receptor and longevity	1.26 × 10^–3^	8.28 × 10^–3^	*IGF1*, *PIK3R1*, *SOD1*, *SOD2*
Glutathione metabolism	1.45 × 10^–3^	8.95 × 10^–3^	*GPX1*, *GSTM1*, *GSTM3*, *GSTO1*, *GSTO2*, *GSTP1*, *GSTT1*
Cytokine–cytokine receptor interaction	1.48 × 10^–3^	8.95 × 10^–3^	*CCL2*, *CCL3*, *CCR2*, *CXCL8*, *FAS*, *IL10*, *IL12A*, *IL12B*, *IL18*, *IL1A*, *IL1B*, *IL23R*, *IL4*, *IL6*, *IL6R*, *NGFR*, *TGFB1*, *TNF*, *VEGFA*
Serotonergic synapse	1.50 × 10^–3^	8.95 × 10^–3^	*ALOX5*, *APP*, *CYP2D6*, *GNB3*, *HTR2A*, *HTR6*, *KCNJ6*, *MAOA*, *PLA2G4A*, *PTGS2*, *SLC6A4*
Antigen processing and presentation	1.63 × 10^–3^	9.53 × 10^–3^	*CTSS*, *HLA-A*, *HLA-DQB1*, *HLA-DRA*, *HLA-DRB1*, *HLA-DRB5*, *HSPA5*, *TAP2*, *TNF*
Drug metabolism—cytochrome P450	1.88 × 10^–3^	1.05 × 10^–2^	*CYP2D6*, *GSTM1*, *GSTM3*, *GSTO1*, *GSTO2*, *GSTP1*, *GSTT1*, *MAOA*
Cell cycle: G1/S check point	2.13 × 10^–3^	1.18 × 10^–2^	*CDK1*, *CDKN2A*, *GSK3B*, *TGFB1*, *TP53*
Fcε RI signaling pathway	2.26 × 10^–3^	1.23 × 10^–2^	*FCER1G*, *GAB2*, *IL4*, *INPP5D*, *PIK3R1*, *PLA2G4A*, *SOS2*, *TNF*
Apoptosis	2.28 × 10^–3^	1.23 × 10^–2^	*FAS*, *IL1A*, *IL1B*, *NGF*, *NTRK1*, *PIK3R1*, *TNF*, *TP53*, *TRAF2*
Role of Erk5 in neuronal survival	2.61 × 10^–3^	1.39 × 10^–2^	*MEF2A*, *MEF2C*, *NTRK1*, *PIK3R1*
Bioactive peptide-induced signaling pathway	2.90 × 10^–3^	1.52 × 10^–2^	*CAMK2D*, *CDK5*, *GNA11*, *MAPT*, *MYLK*, *PTK2B*
Control of skeletal myogenesis by HDAC and calcium/calmodulin-dependent kinase (CaMK)	2.93 × 10^–3^	1.52 × 10^–2^	*IGF1*, *INS*, *MEF2A*, *MEF2C*, *PIK3R1*
Metabolism of xenobiotics by cytochrome P450	3.22 × 10^–3^	1.62 × 10^–2^	*CYP2D6*, *GSTM1*, *GSTM3*, *GSTO1*, *GSTO2*, *GSTP1*, *GSTT1*, *HSD11B1*
Ras-independent pathway in NK cell-mediated cytotoxicity	3.92 × 10^–3^	1.88 × 10^–2^	*HLA-A*, *IL18*, *PIK3R1*, *PTK2B*
Dopaminergic synapse	4.48 × 10^–3^	2.11 × 10^–2^	*CAMK2D*, *CLOCK*, *COMT*, *DRD4*, *GNB3*, *GRIN2B*, *GSK3B*, *KCNJ6*, *MAOA*, *PPP2R2B*, *SLC6A3*
Cholinergic synapse	4.57 × 10^–3^	2.12 × 10^–2^	*CAMK2D*, *CHAT*, *CHRNA3*, *CHRNA4*, *CHRNA7*, *CHRNB2*, *GNA11*, *GNB3*, *KCNJ6*, *PIK3R1*
The co-stimulatory signal during T-cell activation	4.72 × 10^–3^	2.17 × 10^–2^	*HLA-DRA*, *HLA-DRB1*, *LCK*, *PIK3R1*
Adhesion and diapedesis of lymphocytes	5.03 × 10^–3^	2.28 × 10^–2^	*CXCL8*, *ICAM1*, *IL1A*
Notch signaling pathway	5.07 × 10^–3^	2.28 × 10^–2^	*APH1A*, *APH1B*, *NCSTN*, *PSEN1*, *PSEN2*, *PSENEN*
Role of ERBB2 in signal transduction and oncology	5.61 × 10^–3^	2.50 × 10^–2^	*ESR1*, *IL6*, *IL6R*, *PIK3R1*
Aminoacyl-tRNA biosynthesis	6.37 × 10^–3^	2.80 × 10^–2^	*MT-TG*, *MT-TH*, *MT-TL2*, *MT-TQ*, *MT-TR*, *MT-TS2*, *MT-TT*
Trka receptor signaling pathway	6.55 × 10^–3^	2.80 × 10^–2^	*NGF*, *NTRK1*, *PIK3R1*
Rac 1 cell motility signaling pathway	6.62 × 10^–3^	2.80 × 10^–2^	*CDK5*, *CDK5R1*, *MYLK*, *PIK3R1*
CTCF: first multivalent nuclear factor	6.62 × 10^–3^	2.80 × 10^–2^	*CDKN2A*, *PIK3R1*, *TGFB1*, *TP53*
Regulation of PGC-1a	7.74 × 10^–3^	3.21 × 10^–2^	*CAMK2D*, *MEF2A*, *MEF2C*, *PPARA*
Calcium signaling pathway	7.85 × 10^–3^	3.22 × 10^–2^	*ADRB1*, *ADRB2*, *ADRB3*, *CAMK2D*, *CHRNA7*, *GNA11*, *HTR2A*, *HTR6*, *LHCGR*, *MYLK*, *NOS1*, *NOS3*, *PTK2B*
Lck and Fyn tyrosine kinases in initiation of TCR activation	8.30 × 10^–3^	3.38 × 10^–2^	*HLA-DRA*, *HLA-DRB1*, *LCK*
Adipocytokine signaling pathway	8.75 × 10^–3^	3.52 × 10^–2^	*CD36*, *IRS1*, *PCK1*, *PPARA*, *RXRA*, *TNF*, *TRAF2*
Ras signaling pathway	9.43 × 10^–3^	3.76 × 10^–2^	*EFNA5*, *EXOC2*, *FGF1*, *GAB2*, *GNB3*, *GRIN2B*, *IGF1*, *INS*, *NGF*, *NGFR*, *PIK3R1*, *PLA2G3*, *PLA2G4A*, *SOS2*, *VEGFA*
Prolactin signaling pathway	1.02 × 10^–2^	3.96 × 10^–2^	*ESR1*, *ESR2*, *GSK3B*, *INS*, *LHCGR*, *PIK3R1*, *SOS2*
Catecholamine biosynthesis, tyrosine → dopamine → noradrenaline → adrenaline	1.05 × 10^–2^	3.99 × 10^–2^	*DBH*, *PNMT*
Fat digestion and absorption	1.14 × 10^–2^	4.32 × 10^–2^	*ABCA1*, *APOA1*, *APOA4*, *CD36*, *PLA2G3*
Stress induction of HSP regulation	1.26 × 10^–2^	4.63 × 10^–2^	*FAS*, *IL1A*, *TNF*
Regulation of hematopoiesis by cytokines	1.26 × 10^–2^	4.63 × 10^–2^	*CXCL8*, *IL4*, *IL6*
CTL-mediated immune response against target cells	1.26 × 10^–2^	4.63 × 10^–2^	*FAS*, *HLA-A*, *ICAM1*
Osteoclast differentiation	1.32 × 10^–2^	4.81 × 10^–2^	*GAB2*, *IL1A*, *IL1B*, *LCK*, *PIK3R1*, *PPARG*, *TGFB1*, *TNF*, *TRAF2*, *TREM2*

^a^Alzheimer’s disease-related genes gene set

^b^Calculated by Fisher’s exact test

^c^Adjusted by the Benjamini and Hochberg (BH) method

^d^Genes among Alzgset included in the specific pathway

### Crosstalks among significantly enriched pathways

To explore the correlations between the pathways, we implemented a pathway crosstalk analysis for the 68 enriched pathways. Here we assumed that crosstalk existed in a pathway pair if they had a proportion of common genes in Alzgset [34]. There were 41 pathways including six or more members in Alzgset, of which 37 pathways met the criterion for crosstalk analysis; that is, each pathway shared at least two genes with one or more other pathways. All of the pathway pairs (207 crosstalks among 37 pathways) were used for constructing the pathway crosstalk network and the overlap significance of each pathway pair was evaluated based on the average of JC and OC.

**Fig. 1:**
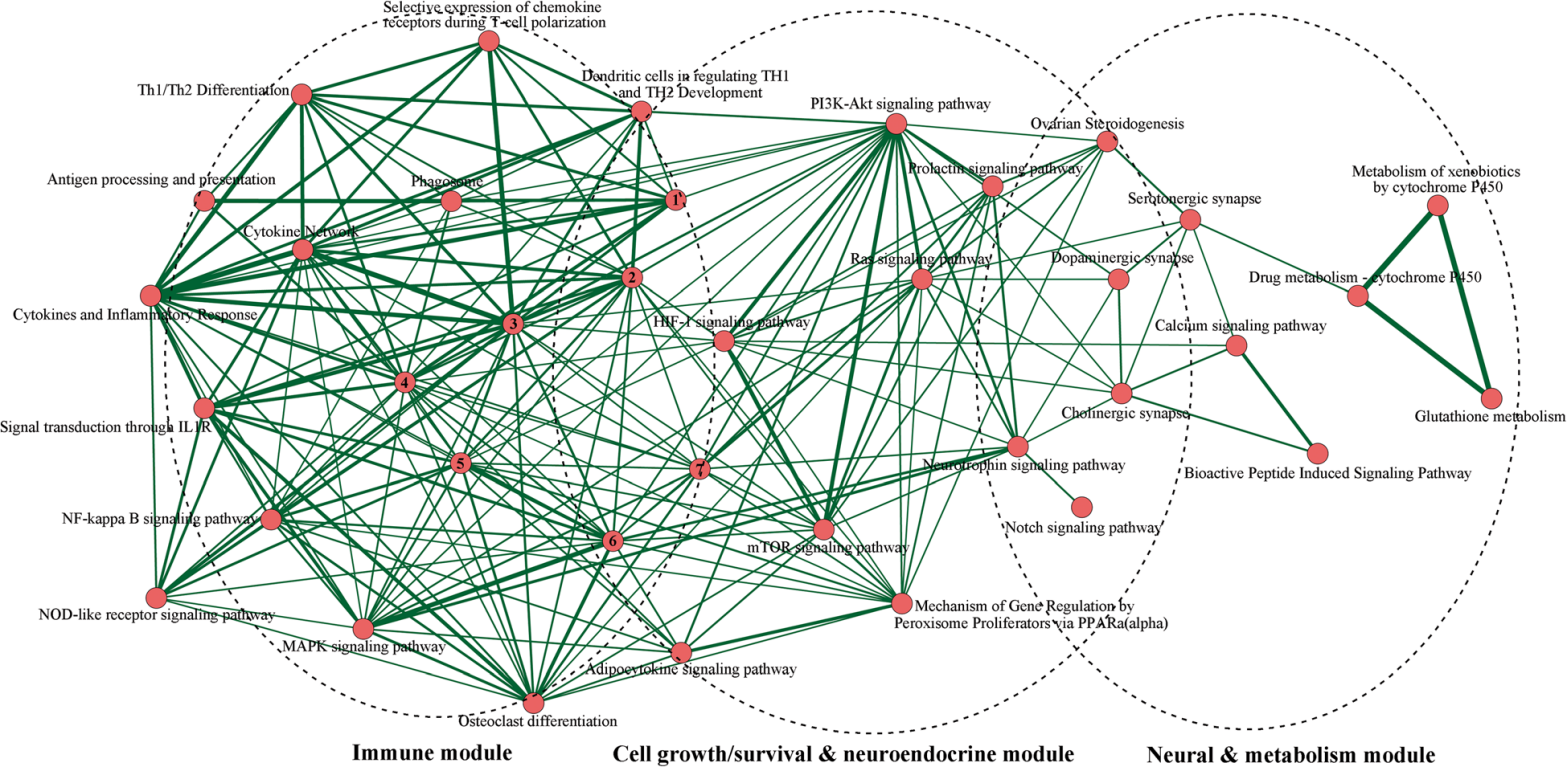
Crosstalk network amid Alzgset-overrepresented pathways. *Vertices*, biological pathways; *lines*, crosstalks among pathways. Width of one line (*edge*) shows direct proportion with the crosstalk level of a given pathway pair. Nodes tagged with numbers represent the following corresponding pathways: *1*, intestinal immune network for IgA production; *2*, toll-like receptor signaling pathway; *3*, cytokine–cytokine receptor interaction; *4*, hematopoietic cell lineage; *5*, TNF signaling pathway; *6*, apoptosis; *7*, Fcε RI signaling pathway

Based on their crosstalks, these pathways could be roughly divided into three major modules, with pathways in each group having more crosstalks with each other than with those outside of this module and more likely being related to the same or similar biological process (Fig. 1). The first module primarily included neuronal-related and xenobiotic or drug metabolism-related pathways (e.g., calcium signaling, dopaminergic synapse, cholinergic synapse, serotonergic synapse and neurotrophin signaling, metabolism of xenobiotics by cytochrome P450, and drug metabolism—cytochrome P450). The major theme of the second module was cell growth/survival and neuroendocrine-related pathways (e.g., PI3K-Akt signaling, mTOR signaling, notch signaling, prolactin signaling, etc.). The third module included immune response-related pathways (e.g., toll-like receptor signaling, Fc epsilon RI signaling pathway). At the same time, the three modules were interlinked with each other, indicating the existence of an AD-specific immune-endocrine-neuronal regulatory network.

### AD-specific protein network

**Fig. 2:**
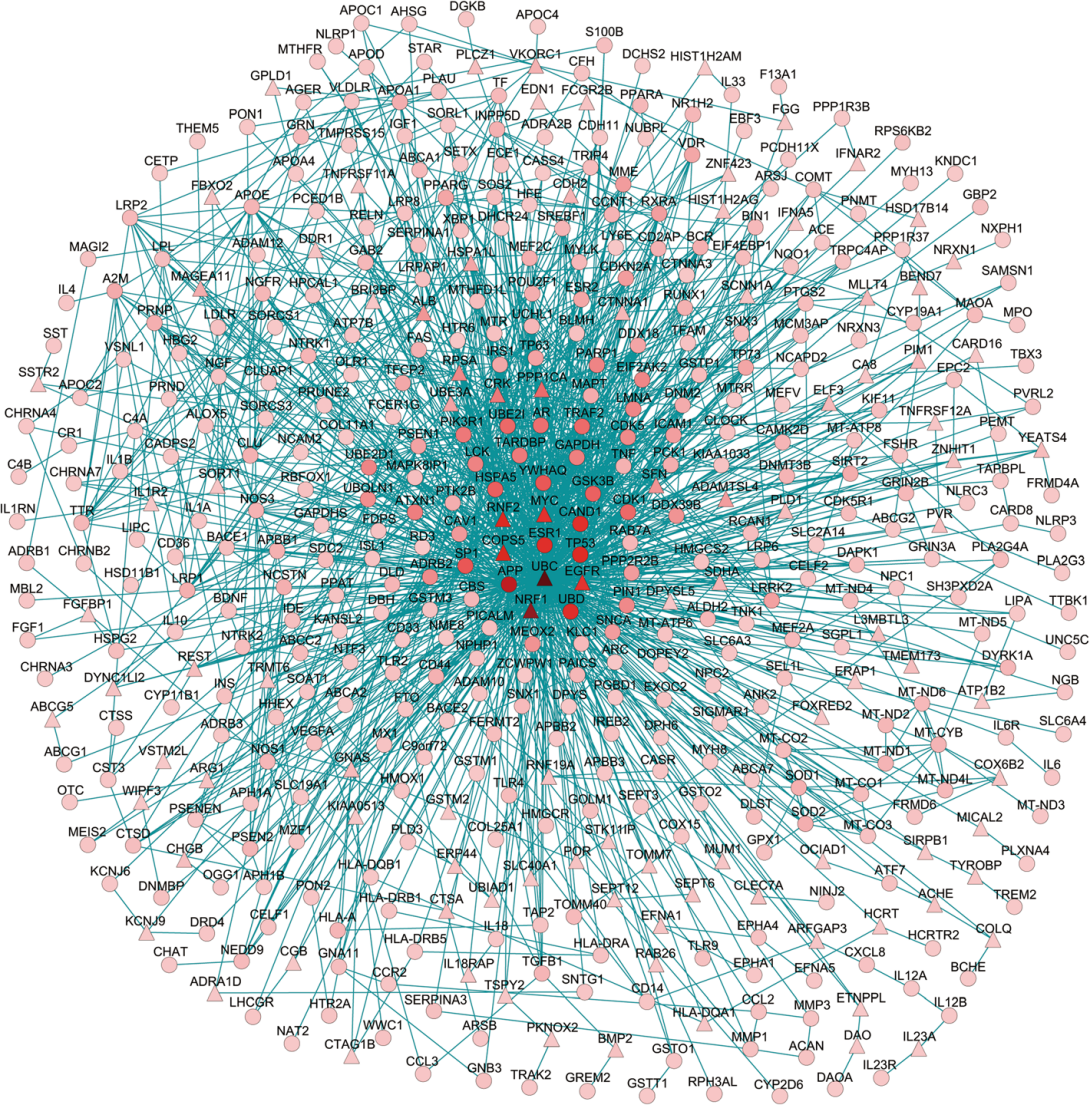
AD-specific protein network built by means of the Steiner minimal tree algorithm, including 496 vertices and 1521 lines. *Circular vertices*, genes of Alzgset; *triangular vertices*, expanding genes. *Color* of a typical vertex designates its corresponding degree under the background of the human protein interactome. *Darkness* of color for a vertex is directly proportional to the corresponding degree value (Color figure online)

To further examine the potential pathological protein network of Alzgset, we constructed a subnetwork for AD from the human protein–protein interaction network via the Steiner minimal tree algorithm. This method tries to connect the largest number of input nodes (genes included in Alzgset in our case) via the least number of interlinking nodes. As shown in Fig. 2, the protein network of AD comprised 496 nodes and 1521 edges (interactions).

As shown, 393 out of 430 Alzgset genes were included in the AD-specific network, which accounted for 79.2% of 496 genes in the network and 91.4% of Alzgset, demonstrating a high coverage of Alzgset in the subnetwork. There were 103 genes in the AD-specific molecular network outside of Alzgset (Table 2). Given that these intermediate genes interacted closely with those known to be related to AD, they might also be involved in the pathological process of the disease phenotype. Notably, a number of the genes—e.g., epidermal growth factor receptor (*EGFR*), nuclear respiratory factor 1 (*NRF1*), somatostatin receptor 2 (*SSTR2*), and sortilin 1 (*SORT1*)—had been shown related to AD in several previous studies [35–38]. Some of these genes have not been reported to be directly involved in the pathophysiological condition of AD, but genes linking to them or other members of the same protein family may have been found to play a role in such processes. For instance, ATP binding cassette subfamily G member 5 (*ABCG5*), a member of a transport system superfamily, involved in ATP binding and transporting of substrates across cytomembranes, was a node in the AD-specific network but was out of Alzgset. However, six members from the same family were included in Alzgset (*ABCA1*, *ABCA2*, *ABCA7*, *ABCC2*, *ABCG1*, and *ABCG2*), and there was experimental evidence for their involvement in AD; for example, the expression reduction or loss of function of *ABCA7* could alter Alzheimer amyloid processing [39]. Solute carrier family 40 member 1 (*SLC40A1*), encoding a cytomembrane protein that may be linked to iron export from duodenal epithelial cells, was also included in the AD-specific network. *SLC40A1*can interact with Golgi membrane protein 1 (*GOLM1*) and hepcidin antimicrobial peptide (*HAMP*). The former was a gene in Alzgset and its mutation may be related to reduced regional gray matter volume in AD patients [40], and the expression of *HAMP* was significantly reduced in hippocampal lysates from AD brains [41]. Thus, it is likely that some of the 103 genes in the AD-specific network may play roles in AD susceptibility and can be novel targets for further exploration.

**Table 2:** Genes included in the AD-specific network but not in Alzgset^a^

Gene symbol	Gene name
*ABCG5*	ATP binding cassette subfamily G member 5
*ACHE*	Acetylcholinesterase (Yt blood group)
*ADAMTSL4*	ADAMTS-like 4
*ADRA1D*	Adrenoceptor alpha 1D
*ALB*	Albumin
*ARFGAP3*	ADP-ribosylation factor GTPase activating protein 3
*ARG1*	Arginase 1
*ATP1B2*	ATPase, Na^+^/K^+^ transporting, beta 2 polypeptide
*BEND7*	BEN domain containing 7
*BMP2*	Bone morphogenetic protein 2
*BRI3BP*	BRI3 binding protein
*CA8*	Carbonic anhydrase VIII
*CARD16*	Caspase recruitment domain family, member 16
*CDH2*	Cadherin 2, type 1, N-cadherin (neuronal)
*CGB*	Chorionic gonadotropin, beta polypeptide
*CHGB*	Chromogranin B
*CLEC7A*	C-type lectin domain family 7, member A
*COLQ*	Collagen-like tail subunit (single strand of homotrimer) of asymmetric acetylcholinesterase
*COPS5*	COP9 signalosome subunit 5
*COX6B2*	Cytochrome c oxidase subunit VIb polypeptide 2 (testis)
*CRK*	V-crk avian sarcoma virus CT10 oncogene homolog
*CTAG1B*	Cancer/testis antigen 1B
*CTNNA1*	Catenin (cadherin-associated protein), alpha 1, 102 kDa
*CTSA*	Cathepsin A
*DAO*	d-amino-acid oxidase
*DDR1*	Discoidin domain receptor tyrosine kinase 1
*DPYSL5*	Dihydropyrimidinase-like 5
*DYNC1LI2*	Dynein, cytoplasmic 1, light intermediate chain 2
*EDN1*	Endothelin 1
*EFNA1*	Ephrin-A1
*EGFR*	Epidermal growth factor receptor
*ELF3*	E74-like factor 3 (ets domain transcription factor, epithelial-specific)
*ERAP1*	Endoplasmic reticulum aminopeptidase 1
*ERP44*	Endoplasmic reticulum protein 44
*ETNPPL*	Ethanolamine-phosphate phospho-lyase
*FBXO2*	F-box protein 2
*FCGR2B*	Fc fragment of IgG, low affinity IIb, receptor (CD32)
*FGFBP1*	Fibroblast growth factor binding protein 1
*FGG*	Fibrinogen gamma chain
*FOXRED2*	FAD-dependent oxidoreductase domain containing 2
*GNAS*	GNAS complex locus
*GPLD1*	Glycosylphosphatidylinositol specific phospholipase D1
*GSTM2*	Glutathione S-transferase mu 2 (muscle)
*HCRT*	Hypocretin (orexin) neuropeptide precursor
*HIST1H2AG*	Histone cluster 1, H2ag
*HIST1H2AM*	Histone cluster 1, H2am
*HLA-DQA1*	Major histocompatibility complex, class II, DQ alpha 1
*HSD17B14*	Hydroxysteroid (17-beta) dehydrogenase 14
*HSPA1L*	Heat shock 70 kDa protein 1-like
*IFNA5*	Interferon, alpha 5
*IFNAR2*	Interferon (alpha, beta and omega) receptor 2
*IL18RAP*	Interleukin-18 receptor accessory protein
*IL1R2*	Interleukin-1 receptor, type II
*IL23A*	Interleukin-23, alpha subunit p19
*KCNJ9*	Potassium channel, inwardly rectifying subfamily J, member 9
*KIAA0513*	KIAA0513
*L3MBTL3*	L(3)mbt-like 3 (Drosophila)
*MAGEA11*	Melanoma antigen family A11
*MICAL2*	Microtubule associated monooxygenase, calponin and LIM domain containing 2
*MLLT4*	Myeloid/lymphoid or mixed-lineage leukemia; translocated to, 4
*MUM1*	Melanoma associated antigen (mutated) 1
*MYC*	V-myc avian myelocytomatosis viral oncogene homolog
*NRF1*	Nuclear respiratory factor 1
*NRXN1*	Neurexin 1
*OCIAD1*	OCIA domain containing 1
*PIM1*	Pim-1 proto-oncogene, serine/threonine kinase
*PKNOX2*	PBX/knotted 1 homeobox 2
*PLCZ1*	Phospholipase C, zeta 1
*PLD1*	Phospholipase D1, phosphatidylcholine-specific
*POR*	P450 (cytochrome) oxidoreductase
*PPP1CA*	Protein phosphatase 1, catalytic subunit, alpha isozyme
*PVR*	Poliovirus receptor
*RAB26*	RAB26, member RAS oncogene family
*REST*	RE1-silencing transcription factor
*RNF19A*	Ring finger protein 19A, RBR E3 ubiquitin protein ligase
*RNF2*	Ring finger protein 2
*RPSA*	Ribosomal protein SA
*SCNN1A*	Sodium channel, non voltage gated 1 alpha subunit
*SDHA*	Succinate dehydrogenase complex, subunit A, flavoprotein (Fp)
*SEPT12*	Septin 12
*SEPT6*	Septin 6
*SFN*	Stratifin
*SIRPB1*	Signal-regulatory protein beta 1
*SLC40A1*	Solute carrier family 40 member 1
*SORT1*	Sortilin 1
*SSTR2*	Somatostatin receptor 2
*STK11IP*	Serine/threonine kinase 11 interacting protein
*TMEM173*	Transmembrane protein 173
*TNFRSF11A*	Tumor necrosis factor receptor superfamily, member 11a, NFKB activator
*TNFRSF12A*	Tumor necrosis factor receptor superfamily, member 12A
*TOMM7*	Translocase of outer mitochondrial membrane 7 homolog (yeast)
*TRMT6*	tRNA methyltransferase 6
*TSPY2*	Testis specific protein, Y-linked 2
*TYROBP*	TYRO protein tyrosine kinase binding protein
*UBC*	Ubiquitin C
*UBE3A*	Ubiquitin protein ligase E3A
*UBIAD1*	UbiA prenyltransferase domain containing 1
*VKORC1*	Vitamin K epoxide reductase complex, subunit 1
*VSTM2L*	V-set and transmembrane domain containing 2 like
*WIPF3*	WAS/WASL interacting protein family, member 3
*YEATS4*	YEATS domain containing 4
*ZNF423*	Zinc finger protein 423
*ZNHIT1*	Zinc finger, HIT-type containing 1

*AD* Alzheimer’s disease

^a^Alzheimer’s disease-related genes gene set

## Discussion

We have made great progress in exploring the molecular mechanisms of Alzheimer’s disease in recent years. With the advancement and maturity of high-throughput technology, we are able to identify the elements related to this disease on much larger scales. Although more and more genes/proteins potentially involved in the disease have been reported, a thorough analysis of the biochemical processes associated with the pathogenesis of AD from the molecular aspect is still missing. In such cases, a systematic analysis of AD-related genes via a pathway-based and network-based analytical framework will provide us with insight into the disease beyond the single candidate gene-based analyses [42–44]. In this study, by pooling and curating human genes related to AD from genetic studies, and systematically delineating the interconnection of these genes by means of pathway-based and network-based analyses, we analyzed AD-related biochemical processes and their interactions.

Compared with the candidate gene(s)-based approach, a comprehensive analysis on AD-related genes conducted in this study has its own advantages. By implementing an extensive compilation and curation of human genes from genetic association studies on AD, we could obtain valuable gene source data for further analysis. Especially, because the risk of AD susceptibility can be attributed to many genes, with multiple genes functioning in a concerted manner and each gene exerting a small effect [45], we took this into consideration by also retrieving genes jointly showing significant genetic association with AD. At the same time, by focusing on the biological correlation of genes, pathway and network analysis can not only give us a more comprehensive view for the pathological mechanisms of AD, but are also more robust to the influence of false-positive genes.

As revealed by function enrichment analysis, genes in Alzgset may play important roles in lipid/lipoprotein-related procedures, the immune system, the metabolic process, drug response processes, and neurodevelopment. For example, terms such as reverse cholesterol transport, positive regulation of interleukin-6 production, response to ethanol, lipoprotein metabolic process, diol metabolic process, xenobiotic metabolic process, and regulation of neuronal synaptic plasticity were overrepresented among Alzgset genes, implying the important roles of these processes in the pathological processes of AD. Furthermore, we noticed several terms of memory, visual learning, social behavior, sleep, axon regeneration, and axon guidance also emerged in the enriched list, concurrent with a-priori biological findings for AD [46–50].

Our biochemical pathway analysis showed that immune-related pathways were enriched among Alzgset, which further highlighted the connections between AD and immune-related biological activities. Previous studies have shown the involvement of neuroinflammation in AD pathology, with inflammatory cytokines exerting central efforts [51, 52]. Simultaneously, four pathways associated with neurotransmitters were found to be overrepresented in Alzgset, coinciding with their essential roles in the etiology and progression of AD. Acetylcholine, dopamine, and serotonin are major neurotransmitters, involved in advanced neuronal functions (e.g., learning, memory, language, etc.), exerting key effects in the pathologic processes of AD. These neurotransmitters could be involved in the damaging procedure of synaptic plasticity like long-term potentiation and long-term depression in AD subjects or animal models, which in turn may impair some synapse-based higher brain functions such as memory and cognition [53–55]. Moreover, our results detected several pathways pertaining to neuroendocrine activities (i.e., ovarian steroidogenesis and prolactin signaling), cuing endocrine processes for the pathogenesis of AD [56, 57]. In addition, the adipocytokine signaling pathway was enriched in Alzgset. Adipocytokines, including leptin, adiponectin, *NAMPT*, *RBP-4*, and other proinflammatory cytokines, have attracted much attention due to their close connection with AD [32, 57, 58]. Detection of the adipocytokine signaling pathway in this study provides further evidence for the relationship between adipocytokine and the development and progression of AD, and may also support the idea that AD could be a metabolic disease [59–61]. As suggested by the results shown, the molecular mechanisms underlying AD are pretty complicated, calling for further thorough studies to decode the underlying pathologic mechanisms.

**Fig. 3:**
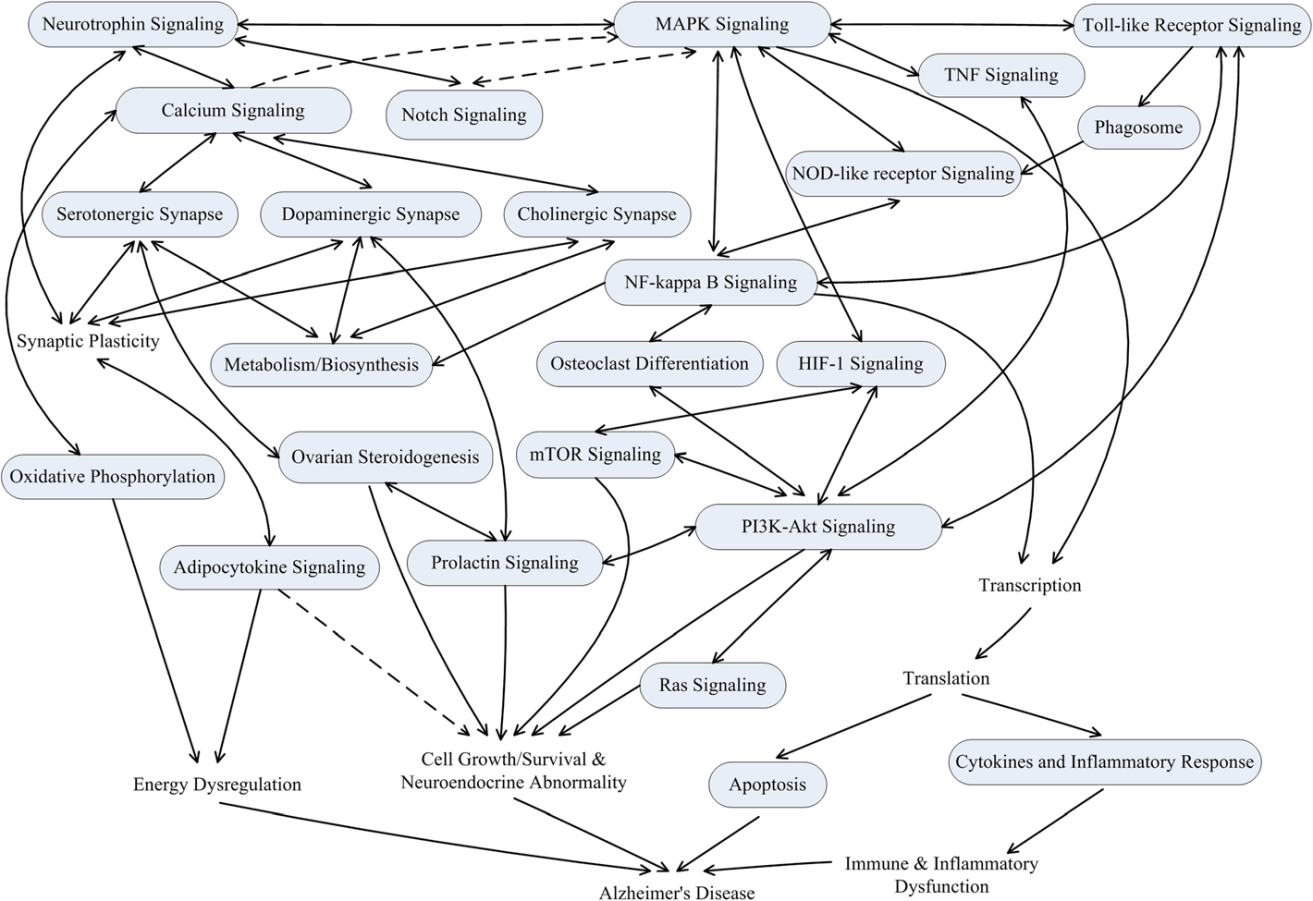
Main biochemical pathways related to AD. Numbers of genetics-based studies have revealed the fact that AD is actually a complex disorder. These major biochemical pathways involved in AD were connected based on their biological relations

Of significance, we detected three major pathway groups through pathway crosstalk analysis. One group basically involved the pathways related to the nervous system and metabolism-related activities. Amid these pathways, cholinergic synapse, the calcium signaling pathway, dopaminergic synapse, serotonergic synapse, and neurotrophin signaling have been well dissected to function in the progress of AD [62–65]. In the second module, pathways were largely dominated by immune response or related functions, and by cell growth/survival and neuroendocrine pathways for the third group. Furthermore, we could notice that these three pathway modules were interconnected and acted as an immune-endocrine-neuronal regulatory network for the AD-related pathological conditions. Of note, one pathway (i.e., intestinal immune network for IgA production) was found to be a component part of the immune module. These results might suggest that the gut–brain axis, made up of immune, neuroendocrine, and neuronal components, was involved in the pathogenesis of AD [66–68], in line with results from pathway crosstalk analysis (i.e., there being three similar modules containing Alzgset-enriched pathways). Subsequently, via in-depth examination, we observed that the immune module has plenty of pathway crosstalks and plenty of crosstalk strength. In turn, the cell growth/survival and neuroendocrine module has lower number and less strength, compared with the immune module; however, in terms of the neural module, the number and strength of crosstalks are greater and larger. In spite of the limited number of crosstalks, there exist paramount crosstalk levels among metabolic pathways. These observed results might provide causal and regulatory hints for AD. Integrating results from biochemical pathway and pathway crosstalk analyses and the a-priori biological knowledge base, the major pathways related to AD could be summarized in a diagram (Fig. 3).

Further, we extracted an AD-specific protein network on the basis of the human protein–protein interaction network. It is worth noting that some linking genes outside Alzgset but included in the human protein–protein interaction network may be potentially related to AD. For example, nuclear respiratory factor-1 (*NRF1*) could be affected by early changes in genes participating in the insulin and energy metabolism pathways in an *APP/PS1* transgenic mouse model of AD [69]. *TYROBP*, a transmembrane signaling protein, appeared in our AD-specific subnetwork. By constructing gene regulatory networks in 1647 postmortem brain tissues from late-onset Alzheimer’s disease (LOAD) patients and normal subjects, an immune and microglia-related module dominated by genes participating in pathogen phagocytosis was identified, with *TYROBP* as a key causal regulator upregulated in LOAD [70]. *CDH2*, a classical cadherin playing roles in the development of the nervous system, was found with the pathogenic copy number variations from 261 early-onset familial Alzheimer’s disease and early/mixed-onset pedigree individuals using high-density DNA microarrays [71]. By applying cell-based studies and *FBXO2* knockout mice, it was found that *FBXO2* could regulate amyloid precursor protein-related activities in the brain and might modulate AD pathogenesis, coupling with our result to consolidate its involvement in AD [72]. Although no evidence indicated that *VSTM2L*, one of the intermediate genes, was directly related to AD, it interacted with ataxin 1 (*ATXN1*) of Alzgset [73], whose biological function is presently unknown, and also might be a secreted antagonist of Humanin (*HN*) [74] which mediated attenuation of AD-related memory impairment and Aβ-induced AD-like pathological changes [75, 76]. As specified by the results detailed, this protein subnetwork predicting approach could not only engender a significant predicted subnetwork of Alzgset for AD, but could also possess the potentiality to detect promising relevant genes.

There have been several available datasets or projects focused on the curation of AD-related genes, including AlzGene [77], Alzheimer’s Disease Neuroimaging Initiative (ADNI) [78], the Alzheimer Disease & Frontotemporal Dementia Mutation Database (AD&FTDMDB) [79], and AlzBase [80]. While AlzGene maintains a comprehensive catalog of genetic association studies on AD and also includes results from meta-analysis of polymorphisms with genotype data available in several GWAS projects on AD, AD&FTDMDB is dedicated to the known mutations of genes associated with AD and frontotemporal dementias from the published reports or presentations at scientific meetings. The ADNI project aims at facilitating the investigation of genetic influences on AD onset and progression reflected in imaging changes, fluid biomarkers, and cognitive status. It has reported several neuroimaging GWAS with imaging quotas as quantitative phenotypes, such as hippocampal volume and hippocampal gray matter density. On the other hand, AlzBase is an integrative database for genes dysregulated in AD and related diseases, and comprises annotations and expression information on more than 7800 differentially expressed genes collected from multiple microarray datasets. These datasets with different features provide valuable information on genes and/or phenotypes for exploring and understanding AD and its mechanisms.

Similar to AlzGene, Alzgset is also a compilation of AD-related genes identified in genetic association studies. While AlzGene includes both genes showing positive and negative association with AD, Alzgset focuses only on the genes reported to be positively associated with AD by the original authors. Because AlzGene has not been updated since April 2011, results from many recent genetic association studies may not be included. In association with studies on candidate genes, some genes may each possess a mild to moderate *p* value, but two or more genes could collectively show a more significant association with AD due to the fact they probably act in a concerted manner. In such cases, all of these candidates were included in Alzgset as long as the original authors could provide sufficient evidence. On the other hand, the genes in AlzGene were selected from meta-analyses for each polymorphism and a relative uniform criterion was adopted, so the genes mentioned may be neglected. Thus, Alzgset should offer an informative supplement for AlzGene and serve as a useful dataset for AD investigation.

However, there were several limitations in this study. First, our pathway-based and network-based analyses results relied on genes in the publications reported to be associated with AD. In view of the fact that identification of risk genes for AD is still an ongoing task, the GO biological process terms, biochemical pathways, and results derived from network analysis should also be treated in the similar manner. Second, we adopted the results and conclusions offered by the original authors of each selected report when collecting the genes, which inevitably impacts our results due to possible bias and insufficiency in the available reports. Then, in order to decrease the false-positive rate of AD-associated genes, we eliminated reports with insignificant or negative results. Nevertheless, we cannot avoid the fact that some genes in those studies might be actually associated with the disease phenotype. Additionally, although the GO terms enriched in Alzgset could provide valuable hints and might serve as an important resource for understanding the molecular mechanisms of AD, it should be noted that GO is biased towards fields like cancer biology and the concepts related to neurology are underrepresented [81]. Thus, some important neurological processes related to AD may be missed in our analysis. At the same time, despite overall levels of protein–protein interaction databases having been greatly improved, the present human interactome is still incomplete and some false-positive data may also be included [82]. Thus, the present research status of the human interactome may also influence our results. It can be expected that, as the protein–protein interaction data become more comprehensive and accurate, the inferred AD-specific subnetwork can become more reliable and valuable.

## Conclusions

In summary, via a systems biology approach, we investigated the pathways and molecular networks related to AD based on the genes associated with the disease. Integrating biological function, biochemical pathway, and pathway crosstalk analyses, we identified that biochemical processes and pathways linked with lipid and/or lipoprotein-related processes, metabolism, the immune system, and neural development were overrepresented among Alzgset and there existed three inter-connected pathway modules: neuronal and metabolic module, cell growth/survival and neuroendocrine clique, and immunological cluster. What is more, an AD-specific protein network was built via the Steiner minimal tree algorithm and some novel genes latently associated with AD were predicted. Such analysis of genes involved in AD will not only improve our understanding of the contribution of genetic factors and their interaction with environmental factors to the pathogenesis of this disease, but will also help us to identify potential biomarkers for further exploration. It could be anticipated that as more genetic factors related to AD are identified, a systematic and comprehensive analysis such as the one adopted in this study will be more useful to explore the molecular mechanisms underlying AD.

## Additional files

Additional file 1: Is a list of the human interactome utilized in this study. The human protein interaction network contains 16,022 genes/proteins and 228,122 interactions. (TXT 5293 kb)

Additional file 2: Table S1. Is presenting a list of genes associated with Alzheimer’s disease and Table S2 presenting the GO biological process terms enriched in Alzgset. (DOC 990 kb)

## Abbreviations

AD: Alzheimer’s disease
Alzgset: Alzheimer’s disease gene set
*APOE*: Apolipoprotein E
*APP*: Amyloid precursor protein
*DYRK1A*: Dual specificity tyrosine-phosphorylation-regulated kinase 1A
FDR: False discovery rate
GO: Gene Ontology
*GSK3B*: Glycogen synthase kinase 3 beta
GWAS: Genome-wide association study
JC: Jaccard coefficient
OC: Overlap coefficient
*PESN1*: Presenilin 1
PINA: Protein interaction network analysis
PPI: Protein–protein interaction
