# Supplementary material for: Analyzing the genes related to Alzheimer’s disease via a network and pathway-based approach
Source: Alzheimers Res Ther. 2017 Apr 27;9:29. doi: 10.1186/s13195-017-0252-z (PMC5406904; doi:10.1186/s13195-017-0252-z)
Supplement: Supplementary file 2 — Is presenting a list of genes associated with Alzheimer’s disease and Table S2 presenting the GO biological process terms enriched in Alzgset. (DOC 990 kb) [file 13195_2017_252_MOESM2_ESM.doc]

**Table S1.** List of genes associated with Alzheimer’s disease

| **Gene symbol** | **Gene name** | **Reference (PMIDs)^*^** |
| --- | --- | --- |
| *A2M* | Alpha-2-macroglobulin | 16784755; 24039871; 20493925; 9697696; 9811940; 10482269; 10527839; 10668709; 10675799; 10976654; 11231028; 12133586; 12221172; 12966032; 15023809; 15931081 |
| *AASDH* | Aminoadipate-semialdehyde dehydrogenase | 22710270 |
| *ABCA1* | ATP-binding cassette, sub-family A (ABC1), member 1 | 24081377; 23181436; 19446537; 20571217; 22377775; 17510949; 17510946; 17324514; 16725228; 15024730; 17335784 |
| *ABCA2* | ATP-binding cassette, sub-family A (ABC1), member 2 | 16752360; 24039871; 15649702 |
| *ABCA7* | ATP-binding cassette, sub-family A (ABC1), member 7 | 23571587; 22832961; 24908168; 21460840; 25807283 |
| *ABCC2* | ATP-binding cassette, sub-family C (CFTR/MRP), member 2 | 23556446 |
| *ABCG1* | ATP-binding cassette, sub-family G (WHITE), member 1 | 17387528 |
| *ABCG2* | ATP-binding cassette, sub-family G (WHITE), member 2 (Junior blood group) | 23827224 |
| *ACAN* | Aggrecan | 17317784 |
| *ACE* | Angiotensin I converting enzyme | 17192785; 25340798; 19105203; 18830724; 16465461; 21533863; 21233092; 20534741; 19539712; 17401152; 9916793; 10567488; 10643899; 10978362; 11015454; 11078932; 11803189; 12668609; 14872014; 15722183; 15832037; 15917098; 16642441 |
| *ADAM10* | ADAM metallopeptidase domain 10 | 21959176 |
| *ADAM12* | ADAM metallopeptidase domain 12 | 17440933 |
| *ADRA2B* | Adrenoceptor alpha 2B | 23499426 |
| *ADRB1* | Adrenoceptor beta 1 | 15212839 |
| *ADRB2* | Adrenoceptor beta 2, surface | 18423577 |
| *ADRB3* | Adrenoceptor beta 3 | 19271249 |
| *AGER* | Advanced glycosylation end product-specific receptor | 20567859; 19902324; 24156267 |
| *AHSG* | Alpha-2-HS-glycoprotein | 16002217 |
| *ALDH2* | Aldehyde dehydrogenase 2 family (mitochondrial) | 21515512; 18201725; 15126281; 10873585 |
| *ALOX5* | Arachidonate 5-lipoxygenase | 20110601 |
| *ANK2* | Ankyrin 2, neuronal | 22710270 |
| *APBB1* | amyloid beta (A4) precursor protein-binding, family B, member 1 (Fe65) | 9799084; 11065130 |
| *APBB2* | Amyloid beta (A4) precursor protein-binding, family B, member 2 | 18852029; 15714520 |
| *APBB3* | Amyloid beta (A4) precursor protein-binding, family B, member 3 | 12402277 |
| *APH1A* | APH1A gamma secretase subunit | 21443683; 19368855 |
| *APH1B* | APH1B gamma secretase subunit | 17466415 |
| *APOA1* | Apolipoprotein A-I | 16130094 |
| *APOA4* | Apolipoprotein A-IV | 16013913; 9272683 |
| *APOC1* | Apolipoprotein C-I | 20145290; 24498013; 19442637; 22832961; 8804993; 24685331; 21533863; 9482248; 9674791; 11702052; 11825674; 15364690; 17998437; 18976728; 19125160 |
| *APOC2* | Apolipoprotein C-II | 19734902; 8128960; 17317784; 19125160 |
| *APOC4* | Apolipoprotein C-IV | 19734902 |
| *APOD* | Apolipoprotein D | 23690001; 18671953; 12497622; 15316799 |
| *APOE* | Apolipoprotein E | 22068907; 24336208; 23990795; 20145290; 19734902; 22832961; 20885792; 23573206; 22005930; 23565137; 20932310; 21123754; 22167654; 23525328; 20100581; 19668339; 19591129; 19442637; 19363267; 18439297; 17498878; 16882736; 16465461; 16399900; 16013913; 15389771; 15201366; 8804993; 24685331; 24650794; 24607147; 24565289; 23887281; 22975751; 22935915; 21825236; 21459483; 20534741; 20061606; 19819468; 18416843; 18317248; 16796589; 8350998; 8103819; 8309625; 8309588; 7473659; 17474819; 17317784; 19118814; 19136949; 20460622; 21390209 |
| *APP* | Amyloid beta (A4) precursor protein | 22801501; 22312439; 17112637 |
| *AR* | Androgen receptor | 12668243 |
| *ARC* | Activity-regulated cytoskeleton-associated protein | 22622366 |
| *ARMS2* | Age-related maculopathy susceptibility 2 | 18688167 |
| *ARSB* | Arylsulfatase B | 19668339 |
| *ARSJ* | Arylsulfatase family, member J | 22710270 |
| *ATF7* | Activating transcription factor 7 | 16770605 |
| *ATP7B* | ATPase, Cu++ transporting, beta polypeptide | 23948886; 22950421; 23830383; 22356903 |
| *ATXN1* | Ataxin 1 | 20308783; 18976728 |
| *BACE1* | Beta-site APP-cleaving enzyme 1 | 17854420; 19462468; 19441127; 12707937; 12824768; 12928915; 14681914; 15784960; 15931081; 18182766 |
| *BACE2* | Beta-site APP-cleaving enzyme 2 | 16023140 |
| *BCHE* | Butyrylcholinesterase | 18334913; 20058037; 16399900; 18290843; 9302273; 9696068; 10399868; 10482954; 10699053; 11015454; 11125748; 15519745; 16020944; 17410321 |
| *BCR* | Breakpoint cluster region | 17317784 |
| *BDNF* | Brain-derived neurotrophic factor | 23215636; 19812463; 19504537; 17293537; 11244490; 11840305; 12192623; 15375678; 15838855; 15896483; 16054753; 16391475 |
| *BIN1* | Bridging integrator 1 | 23570733; 24582639; 23565137; 21460841; 22832961; 23232270; 23571587; 21390209; 21460840; 21220176; 20460622; 21059989 |
| *BLMH* | Bleomycin hydrolase | 10822352; 9500538 |
| *C4A* | Complement component 4A (Rodgers blood group) | 6538270 |
| *C4B* | Complement component 4B (Chido blood group) | 6538270 |
| *C9orf72* | Chromosome 9 open reading frame 72 | 23107433 |
| *CADPS2* | Ca++-dependent secretion activator 2 | 22710270 |
| *CALHM1* | Calcium homeostasis modulator 1 | 20164592; 20061624; 18585350 |
| *CAMK2D* | Calcium/calmodulin-dependent protein kinase II delta | 22710270 |
| *CAND1* | Cullin-associated and neddylation-dissociated 1 | 19668339 |
| *CARD8* | Caspase recruitment domain family, member 8 | 19252766; 18841008 |
| *CASR* | Calcium-sensing receptor | 19035514 |
| *CASS4* | Cas scaffolding protein family member 4 | 24162737 |
| *CAV1* | Caveolin 1, caveolae protein, 22kDa | 19475601 |
| *CBS* | Cystathionine-beta-synthase | 15975077; 16399900 |
| *CCL2* | Chemokine (C-C motif) ligand 2 | 15288699 |
| *CCL3* | Chemokine (C-C motif) ligand 3 | 18242850 |
| *CCNT1* | Cyclin T1 | 16770605 |
| *CCR2* | Chemokine (C-C motif) receptor 2 | 15465089 |
| *CD14* | CD14 molecule | 17900622 |
| *CD2AP* | CD2-associated protein | 21460841; 21460840 |
| *CD33* | CD33 molecule | 21460841; 22167654; 23232270; 23571587; 22382309; 18976728; 21460840 |
| *CD36* | CD36 molecule (thrombospondin receptor) | 15786443 |
| *CD44* | CD44 molecule (Indian blood group) | 22710270 |
| *CDH11* | Cadherin 11, type 2, OB-cadherin (osteoblast) | 22710270 |
| *CDK1* | Cyclin-dependent kinase 1 | 17498878; 21811019; 12648761; 16192727 |
| *CDK5* | Cyclin-dependent kinase 5 | 18350355; 15917097 |
| *CDK5R1* | Cyclin-dependent kinase 5, regulatory subunit 1 (p35) | 19154537 |
| *CDKN2A* | Cyclin-dependent kinase inhibitor 2A | 18761660 |
| *CELF1* | CUGBP, Elav-like family member 1 | 24162737 |
| *CELF2* | CUGBP, Elav-like family member 2 | 21059989; 21379329 |
| *CETP* | Cholesteryl ester transfer protein, plasma | 22122979; 21892657; 25105518; 24468472; 20068209; 16096813; 17503098 |
| *CFH* | Complement factor H | 18163432 |
| *CH25H* | Cholesterol 25-hydroxylase | 16909003 |
| *CHAT* | Choline O-acetyltransferase | 21507424; 18780301; 24039871; 21602657; 12401548; 15690550; 16223550; 16480703 |
| *CHRFAM7A* | CHRNA7 (cholinergic receptor, nicotinic, alpha 7, exons 5-10) and FAM7A (family with sequence similarity 7A, exons A-E) fusion | 23227193; 19641318 |
| *CHRNA3* | Cholinergic receptor, nicotinic, alpha 3 (neuronal) | 12214130 |
| *CHRNA4* | Cholinergic receptor, nicotinic, alpha 4 (neuronal) | 12214130; 16608406 |
| *CHRNA7* | Cholinergic receptor, nicotinic, alpha 7 (neuronal) | 18078695; 18057084 |
| *CHRNB2* | Cholinergic receptor, nicotinic, beta 2 (neuronal) | 17192785; 18830724; 15026168; 20413850 |
| *CLOCK* | Clock circadian regulator | 23912676; 23357097; 23781009 |
| *CLSTN2* | Calsyntenin 2 | 22710270 |
| *CLU* | Clusterin | 21460841; 19734902; 22402018; 22710270; 23411014; 22296908; 22248099; 22122982; 22015308; 21300948; 20697030; 20599866; 20554627; 20534741; 19734903; 20460622; 21059989 |
| *CLUAP1* | Clusterin associated protein 1 | 22710270 |
| *COL11A1* | Collagen, type XI, alpha 1 | 15786443 |
| *COL25A1* | Collagen, type XXV, alpha 1 | 18501477 |
| *COMT* | Catechol-O-methyltransferase | 23034259; 22890094; 19793392; 15488308; 15591802 |
| *COX10* | COX10 heme A:farnesyltransferase cytochrome c oxidase assembly factor | 19826901 |
| *COX15* | Cytochrome c oxidase assembly homolog 15 (yeast) | 19826901 |
| *CR1* | Complement component (3b/4b) receptor 1 (Knops blood group) | 24018213; 21460841; 22402018; 20558149; 22819390; 22960360; 22710270; 23571587; 24996192; 22015308; 21784344; 21459483; 21403675; 21300948; 20697030; 20554627; 20534741; 19734903; 21460840 |
| *CST3* | Cystatin C | 22435454; 17192785; 16399900; 20534741; 17310123; 10993992; 11074789; 11711204; 15728313 |
| *CTNNA3* | Catenin (cadherin-associated protein), alpha 3 | 17761686; 17209133; 14559775; 16199552; 17573676 |
| *CTSD* | Cathepsin D | 19926167; 16784755; 16399900; 20083556; 10218883; 10716266 |
| *CTSS* | Cathepsin S | 17317784 |
| *CXCL8* | Chemokine (C-X-C motif) ligand 8 | 19246914 |
| *CYP11B1* | Cytochrome P450, family 11, subfamily B, polypeptide 1 | 22710270 |
| *CYP19A1* | Cytochrome P450, family 19, subfamily A, polypeptide 1 | 16882736; 20932310; 23635391; 19879925; 18299793; 15079018; 16020944 |
| *CYP2D6* | Cytochrome P450, family 2, subfamily D, polypeptide 6 | 24909950 |
| *CYP46A1* | Cytochrome P450, family 46, subfamily A, polypeptide 1 | 19363267; 23070465; 22528464; 16013913; 20693622; 19286353; 16734927; 12232784; 15165699; 15450677; 16340204 |
| *DAOA* | D-amino acid oxidase activator | 22710270 |
| *DAPK1* | Death-associated protein kinase 1 | 21167819; 16847012 |
| *DBH* | Dopamine beta-hydroxylase (dopamine beta-monooxygenase) | 21070631; 21911036; 16421143 |
| *DCHS2* | Dachsous cadherin-related 2 | 22005931 |
| *DDX18* | DEAD (Asp-Glu-Ala-Asp) box polypeptide 18 | 22710270 |
| *DDX39B* | DEAD (Asp-Glu-Ala-Asp) box polypeptide 39B | 18715507 |
| *DGKB* | Diacylglycerol kinase, beta 90kDa | 21059989 |
| *DHCR24* | 24-dehydrocholesterol reductase | 17510943 |
| *DLD* | Dihydrolipoamide dehydrogenase | 15389771; 17342416 |
| *DLST* | Dihydrolipoamide S-succinyltransferase (E2 component of 2-oxo-glutarate complex) | 10227647; 9365459 |
| *DNM2* | Dynamin 2 | 18236001 |
| *DNMBP* | Dynamin binding protein | 18359537; 16740596 |
| *DNMT3B* | DNA (cytosine-5-)-methyltransferase 3 beta | 25038421 |
| *DOPEY2* | Dopey family member 2 | 23227193 |
| *DPH6* | Diphthamine biosynthesis 6 | 22710270 |
| *DPYS* | Dihydropyrimidinase | 15786443 |
| *DRD4* | Dopamine receptor D4 | 23034259 |
| *DYRK1A* | Dual-specificity tyrosine-(Y)-phosphorylation regulated kinase 1A | 17135279 |
| *EBF3* | Early B-cell factor 3 | 17317784 |
| *ECE1* | Endothelin converting enzyme 1 | 20037208; 18334739; 15340356 |
| *EFNA5* | Ephrin-A5 | 19668339 |
| *EIF2AK2* | Eukaryotic translation initiation factor 2-alpha kinase 2 | 17420072 |
| *EIF4EBP1* | Eukaryotic translation initiation factor 4E binding protein 1 | 19271249 |
| *EPC2* | Enhancer of polycomb homolog 2 (Drosophila) | 21123754 |
| *EPHA1* | EPH receptor A1 | 22832961; 23571587; 21460841; 21460840 |
| *EPHA4* | EPH receptor A4 | 20100581 |
| *ESR1* | Estrogen receptor 1 | 20674091; 24133901; 16914837; 17192785; 24857745; 25061285; 19586561; 17446729; 16796589; 16699281; 10558867; 10713392; 10765041; 11406328; 12852830; 17410321 |
| *ESR2* | Estrogen receptor 2 (ER beta) | 11406328; 11781694; 17132983 |
| *EXOC2* | Exocyst complex component 2 | 22710270 |
| *EXOC3L2* | Exocyst complex component 3-like 2 | 23663385; 20460622 |
| *F13A1* | Coagulation factor XIII, A1 polypeptide | 17288735 |
| *FAS* | Fas cell surface death receptor | 16921240; 11129341; 17573676 |
| *FCER1G* | Fc fragment of IgE, high affinity I, receptor for; gamma polypeptide | 15786443 |
| *FDPS* | Farnesyl diphosphate synthase | 17387528 |
| *FERMT2* | Fermitin family member 2 | 24162737; 24495969 |
| *FGF1* | Fibroblast growth factor 1 (acidic) | 24464990; 15358178 |
| *FRMD4A* | FERM domain containing 4A | 22430674 |
| *FRMD6* | FERM domain containing 6 | 22190428 |
| *FSHR* | Follicle stimulating hormone receptor | 21865747 |
| *FTO* | Fat mass and obesity associated | 23251365 |
| *GAB2* | GRB2-associated binding protein 2 | 23724096; 23525328; 21285854; 19204163; 21108942; 21132329; 22190428; 24161894; 19276544; 19118819; 18853460; 17553421 |
| *GALP* | Galanin-like peptide | 17317784 |
| *GAPDH* | Glyceraldehyde-3-phosphate dehydrogenase | 20864222; 18340469; 16832079; 15507493 |
| *GAPDHS* | Glyceraldehyde-3-phosphate dehydrogenase, spermatogenic | 17192785 |
| *GBP2* | Guanylate binding protein 2, interferon-inducible | 15786443 |
| *GNA11* | Guanine nucleotide binding protein (G protein), alpha 11 (Gq class) | 15786443 |
| *GNB3* | Guanine nucleotide binding protein (G protein), beta polypeptide 3 | 15212839 |
| *GOLM1* | Golgi membrane protein 1 | 22167654; 20592574; 17998437 |
| *GPX1* | Glutathione peroxidase 1 | 20724907 |
| *GREM2* | Gremlin 2, DAN family BMP antagonist | 22710270 |
| *GRIN2B* | Glutamate receptor, ionotropic, N-methyl D-aspartate 2B | 18983893 |
| *GRIN3A* | Glutamate receptor, ionotropic, N-methyl-D-aspartate 3A | 20016182 |
| *GRN* | Granulin | 19016491; 24680777; 21212639; 18565828 |
| *GSK3B* | Glycogen synthase kinase 3 beta | 23525328; 21443865; 18991351; 19154537; 18852354; 16428884 |
| *GSTM1* | Glutathione S-transferase mu 1 | 22381228 |
| *GSTM3* | Glutathione S-transferase mu 3 (brain) | 23036584; 18423940; 17904251 |
| *GSTO1* | Glutathione S-transferase omega 1 | 22100662; 20818931 |
| *GSTO2* | Glutathione S-transferase omega 2 | 22494505 |
| *GSTP1* | Glutathione S-transferase pi 1 | 18298341; 15805147 |
| *GSTT1* | Glutathione S-transferase theta 1 | 22813660; 18298341; 10215103 |
| *HACD1* | 3-hydroxyacyl-CoA dehydratase 1 | 19241460 |
| *HBG2* | Hemoglobin, gamma G | 17157413 |
| *HCRTR2* | Hypocretin (orexin) receptor 2 | 24969517 |
| *HFE* | Hemochromatosis | 21701828; 20029940; 19429178; 10861683; 15060098 |
| *HHEX* | Hematopoietically expressed homeobox | 23036584 |
| *HLA-A* | Major histocompatibility complex, class I, A | 18936542; 16608404; 22710270; 20074462; 11044581 |
| *HLA-DQB1* | Major histocompatibility complex, class II, DQ beta 1 | 25651370 |
| *HLA-DRA* | Major histocompatibility complex, class II, DR alpha | 23227193 |
| *HLA-DRB1* | Major histocompatibility complex, class II, DR beta 1 | 24162737; 25651370; 9172155; 10568518 |
| *HLA-DRB5* | Major histocompatibility complex, class II, DR beta 5 | 24162737 |
| *HMGCR* | 3-hydroxy-3-methylglutaryl-CoA reductase | 19446537 |
| *HMGCS2* | 3-hydroxy-3-methylglutaryl-CoA synthase 2 (mitochondrial) | 17387528 |
| *HMOX1* | Heme oxygenase (decycling) 1 | 18841019; 18597895 |
| *HPCAL1* | Hippocalcin-like 1 | 21059989 |
| *HSD11B1* | Hydroxysteroid (11-beta) dehydrogenase 1 | 14583441 |
| *HSPA5* | Heat shock 70kDa protein 5 (glucose-regulated protein, 78kDa) | 18781276 |
| *HSPG2* | Heparan sulfate proteoglycan 2 | 14625044 |
| *HTR2A* | 5-hydroxytryptamine (serotonin) receptor 2A, G protein-coupled | 23274704; 17183148 |
| *HTR6* | 5-hydroxytryptamine (serotonin) receptor 6, G protein-coupled | 15531082; 10624811 |
| *ICAM1* | Intercellular adhesion molecule 1 | 12498973; 15778814 |
| *IDE* | Insulin-degrading enzyme | 23416320; 23036584; 17192785; 19864659; 22502914; 22107728; 20880607; 18996360; 16876916; 12809979; 14517947; 15024728; 15277615; 17496198; 17573676 |
| *IGBP1P5* | Immunoglobulin (CD79A) binding protein 1 pseudogene 5 | 20100581 |
| *IGF1* | Insulin-like growth factor 1 (somatomedin C) | 21176999; 23089282 |
| *IL10* | Interleukin 10 | 19744138; 23838435; 21911036; 20213229; 19698145; 18299793; 17420099; 14746878; 15212825; 15452323; 15748779 |
| *IL12A* | Interleukin 12A | 25037175 |
| *IL12B* | Interleukin 12B | 25037175 |
| *IL18* | Interleukin 18 | 19073159; 17988833; 17299019 |
| *IL1A* | Interleukin 1, alpha | 23322030; 22513697; 17290104; 15201366; 10716256; 10716257; 10953177; 11065142; 12112093; 12242547; 15465625; 15653174; 16421143 |
| *IL1B* | Interleukin 1, beta | 15201366; 23079713; 22498095; 18717723; 10716257; 10976648; 12112093; 15653174; 16053468; 16226351 |
| *IL1RN* | Interleukin 1 receptor antagonist | 22498095; 15653174; 16226351 |
| *IL23R* | Interleukin 23 receptor | 24703098 |
| *IL33* | Interleukin 33 | 20708824 |
| *IL4* | Interleukin 4 | 24463336; 20213229 |
| *IL6* | Interleukin 6 | 23666170; 23510010; 22015309; 19744138; 19252766; 22701584; 22272811; 21252539; 20667498; 19957198; 19698145; 10319892; 10739887; 11992567; 12352619; 12657090; 12928051; 15212825; 15452323; 15778814; 16421143 |
| *IL6R* | Interleukin 6 receptor | 20197062 |
| *INPP5D* | Inositol polyphosphate-5-phosphatase, 145kDa | 24162737; 24495969 |
| *INS* | Insulin | 22065208; 12938026 |
| *IREB2* | Iron-responsive element binding protein 2 | 16914832 |
| *IRS1* | Insulin receptor substrate 1 | 24589556 |
| *ISL1* | ISL LIM homeobox 1 | 22710270 |
| *KANSL2* | KAT8 regulatory NSL complex subunit 2 | 16770605 |
| *KCNJ6* | Potassium channel, inwardly rectifying subfamily J, member 6 | 17135279 |
| *KIAA1033* | KIAA1033 | 22673115 |
| *KIF11* | Kinesin family member 11 | 23036584 |
| *KLC1* | Kinesin light chain 1 | 17611642; 19911314; 15364413 |
| *KNDC1* | Kinase non-catalytic C-lobe domain (KIND) containing 1 | 22710270 |
| *LCK* | LCK proto-oncogene, Src family tyrosine kinase | 16109429 |
| *LDLR* | Low density lipoprotein receptor | 17239995; 15585340; 15689450; 16311892; 18065781 |
| *LHCGR* | Luteinizing hormone/choriogonadotropin receptor | 18439297 |
| *LIPA* | Lipase A, lysosomal acid, cholesterol esterase | 16013913 |
| *LIPC* | lipase, hepatic | 23181436 |
| *LMNA* | Lamin A/C | 17317784 |
| *LOC642487* | H1 histone family, member O, oocyte-specific pseudogene | 20100581 |
| *LPL* | Lipoprotein lipase | 16965549; 24039871; 16013913; 10206232; 15331147 |
| *LRP1* | Low density lipoprotein receptor-related protein 1 | 23186781; 19684401; 18706476; 9148246; 9222170; 9633759; 9637403; 10394937; 11076057; 10369887; 12898587; 12901493; 16040006; 16311892 |
| *LRP2* | Low density lipoprotein receptor-related protein 2 | 20971101; 20052685 |
| *LRP6* | Low density lipoprotein receptor-related protein 6 | 17517621; 19001172 |
| *LRP8* | Low density lipoprotein receptor-related protein 8, apolipoprotein e receptor | 17614163; 12399018 |
| *LRPAP1* | Low density lipoprotein receptor-related protein associated protein 1 | 11425005 |
| *LRRK2* | Leucine-rich repeat kinase 2 | 23421816; 20018409 |
| *LRRTM3* | Leucine rich repeat transmembrane neuronal 3 | 22393166; 24463050; 17573676 |
| *LY6E* | Lymphocyte antigen 6 complex, locus E | 22710270 |
| *MAGI2* | Membrane associated guanylate kinase, WW and PDZ domain containing 2 | 19668339 |
| *MALRD1* | MAM and LDL receptor class A domain containing 1 | 19591129; 10961670; 11443525 |
| *MAOA* | Monoamine oxidase A | 12098640; 16186632 |
| *MAPK8IP1* | Mitogen-activated protein kinase 8 interacting protein 1 | 17614163 |
| *MAPT* | Microtubule-associated protein tau | 21443865; 24923570; 19684401; 19091059; 18991351; 18841019; 22556362; 22176350; 22027014; 19070646; 18319590; 10643798; 15106853; 16000317 |
| *MBL2* | Mannose-binding lectin (protein C) 2, soluble | 23348713 |
| *MCM3AP* | Minichromosome maintenance complex component 3 associated protein | 15786443 |
| *MEF2A* | Myocyte enhancer factor 2A | 17112666 |
| *MEF2C* | Myocyte enhancer factor 2C | 24162737; 24495969 |
| *MEFV* | Mediterranean fever | 17090974 |
| *MEIS2* | Meis homeobox 2 | 22710270 |
| *MEOX2* | Mesenchyme homeobox 2 | 22710270 |
| *MIR146A* | MicroRNA 146a | 24586483 |
| *MME* | Membrane metallo-endopeptidase | 19864659; 17928142; 12768360; 14739539; 15548496; 15860464 |
| *MMP1* | Matrix metallopeptidase 1 | 17077200 |
| *MMP3* | Matrix metallopeptidase 3 | 15337261; 25340798; 17077200 |
| *MPO* | Myeloperoxidase | 9918702; 11087769; 11161635; 12946561; 15023809 |
| *MS4A4A* | Membrane-spanning 4-domains, subfamily A, member 4A | 21460841; 22832961 |
| *MS4A4E* | Membrane-spanning 4-domains, subfamily A, member 4E | 21460840 |
| *MS4A6A* | Membrane-spanning 4-domains, subfamily A, member 6A | 24064185; 23232270; 21460840; 22382309 |
| *MS4A6E* | Membrane-spanning 4-domains, subfamily A, member 6E | 22832961 |
| *MT-ATP6* | Mitochondrially encoded ATP synthase 6 | 7835898 |
| *MT-ATP8* | Mitochondrially encoded ATP synthase 8 | 7835898 |
| *MT-CO3* | Mitochondrially encoded cytochrome c oxidase III | 7835898; 16358358; 23028804; 20538375 |
| *MT-TG* | Mitochondrially encoded tRNA glycine | 7835898 |
| *MT-ND3* | Mitochondrially encoded NADH dehydrogenase 3 | 7835898; 15234467 |
| *MT-TR* | Mitochondrially encoded tRNA arginine | 7835898 |
| *MT-ND4L* | Mitochondrially encoded NADH 4L dehydrogenase | 7835898; 10377009 |
| *MT-ND4* | Mitochondrially encoded NADH dehydrogenase 4 | 7835898; 16920408; 20538375 |
| *MT-TH* | Mitochondrially encoded tRNA histidine | 7835898 |
| *MT-TS2* | Mitochondrially encoded tRNA serine 2 (AGU/C) | 7835898 |
| *MT-TL2* | Mitochondrially encoded tRNA leucine 2 (CUN) | 7835898; 10377009; 20538375 |
| *MT-ND5* | Mitochondrially encoded NADH dehydrogenase 5 | 7835898; 16920408; 20538375 |
| *MT-ND6* | Mitochondrially encoded NADH dehydrogenase 6 | 16920408 |
| *MT-CYB* | Mitochondrially encoded cytochrome b | 10377009 |
| *MT-TT* | Mitochondrially encoded tRNA threonine | 10377009 |
| *MT-RNR1* | Mitochondrially encoded 12S RNA | 10377009; 19703591 |
| *MT-ND1* | Mitochondrially encoded NADH dehydrogenase 1 | 16920408; 23028804 |
| *MT-TQ* | Mitochondrially encoded tRNA glutamine | 9292870; 7624338; 8104867; 19703591; 20700462 |
| *MT-ND2* | Mitochondrially encoded NADH dehydrogenase 2 | 1370613; 16920408; 23028804 |
| *MT-CO1* | Mitochondrially encoded cytochrome c oxidase I | 16920408; 9114023; 21822896 |
| *MT-CO2* | Mitochondrially encoded cytochrome c oxidase II | 9114023; 16920408 |
| *MTHFD1L* | Methylenetetrahydrofolate dehydrogenase (NADP+ dependent) 1-like | 22330827; 21741665; 20885792 |
| *MTHFR* | Methylenetetrahydrofolate reductase (NAD(P)H) | 24223459; 23659764; 22015309; 17192785; 25486592; 22034983; 19246914; 21663380; 20600372; 16906459; 15123333; 16055944;  18258338 |
| *MTR* | 5-methyltetrahydrofolate-homocysteine methyltransferase | 16399900; 12876480 |
| *MTRR* | 5-methyltetrahydrofolate-homocysteine methyltransferase reductase | 22034983 |
| *MX1* | MX dynamin-like GTPase 1 | 22950423 |
| *MYH13* | Myosin, heavy chain 13, skeletal muscle | 17317784 |
| *MYH8* | Myosin, heavy chain 8, skeletal muscle, perinatal | 15786443 |
| *MYLK* | Myosin light chain kinase | 22710270 |
| *MZF1* | Myeloid zinc finger 1 | 23241556 |
| *NAT2* | N-acetyltransferase 2 (arylamine N-acetyltransferase) | 10208637; 15339384 |
| *NCAM2* | Neural cell adhesion molecule 2 | 20932310; 17135279 |
| *NCAPD2* | Non-SMC condensin I complex, subunit D2 | 19451718; 18340469 |
| *NCSTN* | Nicastrin | 19840113; 19394408; 17192785; 21364883; 11992262; 15249634; 16423463 |
| *NEDD9* | Neural precursor cell expressed, developmentally down-regulated 9 | 22963925; 21399483; 21059344; 20430066; 18063669 |
| *NGB* | Neuroglobin | 19010568 |
| *NGF* | Nerve growth factor (beta polypeptide) | 22330829 |
| *NGFR* | Nerve growth factor receptor | 22236693; 18780967 |
| *NINJ2* | Ninjurin 2 | 21674003 |
| *NLRC3* | NLR family, CARD domain containing 3 | 22710270 |
| *NLRP1* | NLR family, pyrin domain containing 1 | 21946017 |
| *NLRP3* | NLR family, pyrin domain containing 3 | 24144834 |
| *NME8* | NME/NM23 family member 8 | 24162737 |
| *NOS1* | Nitric oxide synthase 1 (neuronal) | 21098972; 17418914; 15765269 |
| *NOS3* | Nitric oxide synthase 3 (endothelial cell) | 20505439; 18183499; 16813604; 10514107 |
| *NPC1* | Niemann-Pick disease, type C1 | 24064683; 20571217; 18834923 |
| *NPC2* | Niemann-Pick disease, type C2 | 17387528 |
| *NPHP1* | Nephronophthisis 1 (juvenile) | 22710270 |
| *NQO1* | NAD(P)H dehydrogenase, quinone 1 | 18253865 |
| *NR1H2* | Nuclear receptor subfamily 1, group H, member 2 | 17900622; 16207502; 18597895 |
| *NRXN3* | Neurexin 3 | 23403532 |
| *NTF3* | Neurotrophin 3 | 9502217 |
| *NTRK1* | Neurotrophic tyrosine kinase, receptor, type 1 | 18780967 |
| *NTRK2* | Neurotrophic tyrosine kinase, receptor, type 2 | 17918233; 18780967 |
| *NUBPL* | Nucleotide binding protein-like | 22710270 |
| *NXPH1* | Neurexophilin 1 | 20100581 |
| *OGFRL1* | Opioid growth factor receptor-like 1 | 22710270 |
| *OGG1* | 8-oxoguanine DNA glycosylase | 19630534 |
| *OLR1* | Oxidized low density lipoprotein (lectin-like) receptor 1 | 21709374; 17854420; 16013913; 12384789; 12807963; 15860461; 18191876 |
| *OTC* | Ornithine carbamoyltransferase | 18983895; 17893704 |
| *PAICS* | Phosphoribosylaminoimidazole carboxylase, phosphoribosylaminoimidazole succinocarboxamide synthetase | 22710270 |
| *PARP1* | Poly (ADP-ribose) polymerase 1 | 20486200; 17290104 |
| *PCDH11X* | Protocadherin 11 X-linked | 19136949 |
| *PCED1B* | PC-esterase domain containing 1B | 19118814 |
| *PCK1* | Phosphoenolpyruvate carboxykinase 1 (soluble) | 17440948; 17317784 |
| *PEMT* | Phosphatidylethanolamine N-methyltransferase | 21881829 |
| *PGBD1* | PiggyBac transposable element derived 1 | 17317784 |
| *PICALM* | Phosphatidylinositol binding clathrin assembly protein | 23572399; 23565137; 21460841; 22402018; 19734902; 22832961; 22975751; 22935915; 22015308; 21459483; 21300948; 21220176; 20697030; 20554627; 20534741; 20460622 |
| *PIK3R1* | Phosphoinositide-3-kinase, regulatory subunit 1 (alpha) | 12185156 |
| *PIN1* | Peptidylprolyl cis/trans isomerase, NIMA-interacting 1 | 16384626 |
| *PLA2G3* | Phospholipase A2, group III | 20930276 |
| *PLA2G4A* | Phospholipase A2, group IVA (cytosolic, calcium-dependent) | 20464283 |
| *PLAU* | Plasminogen activator, urokinase | 23813610; 17174555; 16967469; 16825285; 12898287; 15615772; 17573676 |
| *PLD3* | Phospholipase D family, member 3 | 24336208 |
| *PLXNA4* | Plexin A4 | 25043464 |
| *PNMT* | Phenylethanolamine N-methyltransferase | 11378842 |
| *PON1* | Paraoxonase 1 | 16319130; 19863653; 18322397; 17624629; 16863614; 12618290 |
| *PON2* | Paraoxonase 2 | 16319130; 14741412; 11803456 |
| *PON3* | Paraoxonase 3 | 16319130 |
| *POU2F1* | POU class 2 homeobox 1 | 15786443 |
| *PPARA* | Peroxisome proliferator-activated receptor alpha | 22065208; 17270153; 12938026 |
| *PPARG* | Peroxisome proliferator-activated receptor gamma | 19660836; 17440948 |
| *PPAT* | phosphoribosyl pyrophosphate amidotransferase | 22710270 |
| *PPP1R37* | Protein phosphatase 1, regulatory subunit 37 | 19734902; 19136949 |
| *PPP1R3B* | Protein phosphatase 1, regulatory subunit 3B | 12185156 |
| *PPP2R2B* | Protein phosphatase 2, regulatory subunit B, beta | 21029765 |
| *PRND* | Prion protein 2 (dublet) | 19363267 |
| *PRNP* | Prion protein | 23399523; 17192785; 19363267; 21799773; 16897605; 12601712; 14745079; 15277640 |
| *PRUNE2* | Prune homolog 2 (Drosophila) | 19668339 |
| *PSEN1* | Presenilin 1 | 23990795; 17192785; 22312439; 18957849; 17719017; 16938285; 10482954; 10573013; 10643802; 10655540; 11389157; 8596269; 8930979; 8947284; 9180219; 9185685; 9270069; 9749607; 12192622; 12413003; 17627113; 19001172 |
| *PSEN2* | Presenilin 2 | 22580083; 22312439; 17727891; 16170650 |
| *PSENEN* | Presenilin enhancer gamma secretase subunit | 17280645; 16423463 |
| *PTGS2* | Prostaglandin-endoperoxide synthase 2 (prostaglandin G/H synthase and cyclooxygenase) | 17234302; 20808133; 22622868; 20110601; 16309832 |
| *PTK2B* | Protein tyrosine kinase 2 beta | 24162737 |
| *PVRL2* | Poliovirus receptor-related 2 (herpesvirus entry mediator B) | 22159054; 19442637; 20885792; 22190428; 22005930; 23565137; 19734902; 19136949 |
| *RAB7A* | RAB7A, member RAS oncogene family | 22673115 |
| [*RBFOX1*](http://www.ncbi.nlm.nih.gov/gene/54715) | RNA binding protein, fox-1 homolog (C. elegans) 1 | 22710270 |
| *RCAN1* | Regulator of calcineurin 1 | 21838211 |
| *RD3* | Retinal degeneration 3 | 22710270 |
| *RELN* | Reelin | 23227193; 18599960 |
| *RPH3AL* | Rabphilin 3A-like (without C2 domains) | 22710270 |
| *RPL7P59* | Ribosomal protein L7 pseudogene 59 | 20100581 |
| *RPS6KB2* | Ribosomal protein S6 kinase, 70kDa, polypeptide 2 | 21811019 |
| *RUNX1* | Runt-related transcription factor 1 | 17135279 |
| *RXRA* | Retinoid X receptor, alpha | 19374686 |
| *S100B* | S100 calcium binding protein B | 17579612 |
| *SAMSN1* | SAM domain, SH3 domain and nuclear localization signals 1 | 17135279 |
| *SDC2* | Syndecan 2 | 22710270 |
| *SEL1L* | Sel-1 suppressor of lin-12-like (C. elegans) | 16412574 |
| *SEPT3* | Septin 3 | 15200238 |
| *SERPINA1* | Serpin peptidase inhibitor, clade A (alpha-1 antiproteinase, antitrypsin), member 1 | 8931713 |
| *SERPINA3* | Serpin peptidase inhibitor, clade A (alpha-1 antiproteinase, antitrypsin), member 3 | 22272609; 18078695; 22294107; 10462111; 9013407; 9003488; 9225693; 9486483; 9749607; 10976648; 11941486; 15653173 |
| *SETX* | Senataxin | 24694197 |
| *SGPL1* | Sphingosine-1-phosphate lyase 1 | 17373700 |
| *SH3PXD2A* | SH3 and PX domains 2A | 17440933 |
| *SIGMAR1* | Sigma non-opioid intracellular receptor 1 | 22561649; 16319298 |
| *SIRT2* | Sirtuin 2 | 22651940; 24497179 |
| *SLC19A1* | Solute carrier family 19 (folate transporter), member 1 | 18258338 |
| *SLC24A4* | Solute carrier family 24 (sodium/potassium/calcium exchanger), member 4 | 24162737 |
| *SLC2A14* | Solute carrier family 2 (facilitated glucose transporter), member 14 | 22421804 |
| *SLC2A9* | Solute carrier family 2 (facilitated glucose transporter), member 9 | 22005930 |
| *SLC6A3* | Solute carrier family 6 (neurotransmitter transporter), member 3 | 23034259 |
| *SLC6A4* | Solute carrier family 6 (neurotransmitter transporter), member 4 | 23274704; 20852909; 17183148; 9106747; 9774779; 11044587; 16186632 |
| *SNCA* | Synuclein, alpha (non A4 component of amyloid precursor) | 11173882 |
| *SNTG1* | Syntrophin, gamma 1 | 22710270 |
| *SNX1* | Sorting nexin 1 | 22673115 |
| *SNX3* | Sorting nexin 3 | 22673115 |
| *SOAT1* | Sterol O-acyltransferase 1 | 16013913; 12851640 |
| *SOD1* | Superoxide dismutase 1, soluble | 25440013 |
| *SOD2* | Superoxide dismutase 2, mitochondrial | 17376152 |
| *SORCS1* | Sortilin-related VPS10 domain containing receptor 1 | 23700427; 23673467; 22353753; 21280075; 23279143; 19241460 |
| *SORCS2* | Sortilin-related VPS10 domain containing receptor 2 | 23673467 |
| *SORCS3* | Sortilin-related VPS10 domain containing receptor 3 | 23673467 |
| *SORL1* | Sortilin-related receptor, L(DLR class) A repeats containing | 23948893; 24938503; 24486888; 23565137; 23525328; 23455993; 24162737; 23673467; 25450149; 25382023; 21220680; 19822782; 19539718; 19368828; 18407551; 17420311; 17220890; 18063222; 18090307; 19001172 |
| *SOS2* | Son of sevenless homolog 2 (Drosophila) | 17440948 |
| *SP1* | Sp1 transcription factor | 23435408 |
| *SREBF1* | Sterol regulatory element binding transcription factor 1 | 15286454 |
| *SST* | Somatostatin | 19733630; 17987251 |
| *STAR* | Steroidogenic acute regulatory protein | 19271249 |
| *STH* | Saitohin | 20852909; 12032355; 12826738; 15136700; 16428884 |
| *TAP2* | Transporter 2, ATP-binding cassette, sub-family B (MDR/TAP) | 16595160 |
| *TAPBPL* | TAP binding protein-like | 18340469 |
| *TARDBP* | TAR DNA binding protein | 19851068 |
| *TBX3* | T-box 3 | 22710270 |
| *TF* | Transferrin | 23968943; 20029940; 17192785; 18830724; 14757931; 8219026; 9402955; 12951205; 15060098 |
| *TFAM* | Transcription factor A, mitochondrial | 20977898; 18430995; 17537576; 17192785; 15464268; 20413850 |
| *TFCP2* | Transcription factor CP2 | 16272261; 11001930; 11283204; 12555245 |
| *TGFB1* | Transforming growth factor, beta 1 | 16082716; 21924590; 17889927; 10914688 |
| *THEM5* | Thioesterase superfamily member 5 | 17317784 |
| *TLR2* | Toll-like receptor 2 | 21989233; 21163333 |
| *TLR4* | Toll-like receptor 4 | 22272615; 21236243; 19006850; 23272070; 16157451 |
| *TLR9* | Toll-like receptor 9 | 23957925 |
| *TMPRSS15* | Transmembrane protease, serine 15 | 17135279 |
| *TNF* | Tumor necrosis factor | 19744138; 19445962; 17192785; 18715507; 24156267; 20693638; 18992723; 18396294; 11121190; 11273064; 12962917; 14745077; 15895461; 16908746 |
| *TNK1* | Tyrosine kinase, non-receptor, 1 | 21132329; 17317784 |
| *TOMM40* | Translocase of outer mitochondrial membrane 40 homolog (yeast) | 23546992; 19442637; 21123754; 23573206; 22005930; 22832961; 23565137; 19734902; 20885792; 20100581; 19668339; 24685331; 23288655; 22008263; 21825236; 21459483; 17317784; 19125160; 19136949 |
| *TP53* | Tumor protein p53 | 19657586 |
| *TP63* | Tumor protein p63 | 20100581 |
| *TP73* | Tumor protein p73 | 19657586; 15175114 |
| *TRAF2* | TNF receptor-associated factor 2 | 18069092 |
| *TRAK2* | Trafficking protein, kinesin binding 2 | 17317784 |
| *TREM2* | Triggering receptor expressed on myeloid cells 2 | 24041969; 23150934; 23855982; 23391427; 25186855; 24439484; 24899047; 23150908 |
| *TREML2* | Triggering receptor expressed on myeloid cells-like 2 | 24439484 |
| *TRIP4* | Thyroid hormone receptor interactor 4 | 24495969 |
| *TRPC4AP* | Transient receptor potential cation channel, subfamily C, member 4 associated protein | 18449908; 19059308 |
| *TTBK1* | Tau tubulin kinase 1 | 21219968; 20096481 |
| *TTR* | Transthyretin | 19328595 |
| *UBD* | Ubiquitin D | 17317784 |
| *UBE2D1* | Ubiquitin-conjugating enzyme E2D 1 | 20100581 |
| *UBE2I* | Ubiquitin-conjugating enzyme E2I | 19765634 |
| *UBQLN1* | Ubiquilin 1 | 25387430; 15745979; 16302009 |
| *UCHL1* | Ubiquitin carboxyl-terminal esterase L1 (ubiquitin thiolesterase) | 16626667 |
| *UNC5C* | Unc-5 netrin receptor C | 25419706 |
| *VDR* | Vitamin D (1,25- dihydroxyvitamin D3) receptor | 23076256; 22306846; 17592215; 21911036 |
| *VEGFA* | Vascular endothelial growth factor A | 23575378; 20138124; 19429179; 19272614; 15732116 |
| *VLDLR* | Very low density lipoprotein receptor | 17854420; 7550352; 9876967; 10206233 |
| *VSNL1* | Visinin-like 1 | 22005930 |
| *WWC1* | WW and C2 domain containing 1 | 24190487; 21185624; 18789830; 17707552 |
| *XBP1* | X-box binding protein 1 | 23421912 |
| *YWHAQ* | Tyrosine 3-monooxygenase/tryptophan 5-monooxygenase activation protein, theta | 18319590; 18290843 |
| *ZCWPW1* | Zinc finger, CW type with PWWP domain 1 | 24162737; 24495969 |
| *ZNF628* | Zinc finger protein 628 | 25329708 |

^*^ References are designated by PMIDs, which are the identifiers of PubMed. PMIDs in each gene term are equal and there’re not priorities in the permutation of the corresponding PMIDs of specified genes.

**Table S2.** Gene Ontology Biological Process terms enriched in Alzgset^a^

| **GO Biological Process Terms^b^** | | **No. of genes^c^** | **P-value^d^** | **P_BH_-value^e^** |
| --- | --- | --- | --- | --- |
| GO:0042493 | response to drug | 56 | 2.63🞨10^-26^ | 4.34🞨10^-24^ |
| GO:0045471 | response to ethanol | 23 | 3.45🞨10^-15^ | 4.62🞨10^-14^ |
| GO:0007613 | memory | 18 | 3.47🞨10^-13^ | 3.51🞨10^-12^ |
| GO:0032755 | positive regulation of interleukin-6 production | 12 | 2.78🞨10^-11^ | 2.19🞨10^-10^ |
| GO:0033700 | phospholipid efflux | 8 | 4.13🞨10^-11^ | 3.19🞨10^-10^ |
| GO:0043691 | reverse cholesterol transport | 9 | 1.43🞨10^-10^ | 1.00🞨10^-9^ |
| GO:0055093 | response to hyperoxia | 10 | 1.58🞨10^-10^ | 1.08🞨10^-9^ |
| GO:0006509 | membrane protein ectodomain proteolysis | 12 | 1.88🞨10^-10^ | 1.26🞨10^-9^ |
| GO:0042632 | cholesterol homeostasis | 14 | 6.38🞨10^-10^ | 3.90🞨10^-9^ |
| GO:0042157 | lipoprotein metabolic process | 17 | 1.87🞨10^-9^ | 1.05🞨10^-8^ |
| GO:0034372 | very-low-density lipoprotein particle remodeling | 7 | 3.04🞨10^-9^ | 1.61🞨10^-8^ |
| GO:0042987 | amyloid precursor protein catabolic process | 7 | 3.04🞨10^-9^ | 1.61🞨10^-8^ |
| GO:0003001 | generation of a signal involved in cell-cell signaling | 31 | 4.54🞨10^-9^ | 2.29🞨10^-8^ |
| GO:0050728 | negative regulation of inflammatory response | 14 | 5.34🞨10^-9^ | 2.64🞨10^-8^ |
| GO:0030168 | platelet activation | 24 | 5.60🞨10^-9^ | 2.76🞨10^-8^ |
| GO:0045429 | positive regulation of nitric oxide biosynthetic process | 10 | 7.14🞨10^-9^ | 3.43🞨10^-8^ |
| GO:0046688 | response to copper ion | 9 | 9.92🞨10^-9^ | 4.71🞨10^-8^ |
| GO:0032760 | positive regulation of tumor necrosis factor production | 10 | 1.00🞨10^-8^ | 4.74🞨10^-8^ |
| GO:0006123 | mitochondrial electron transport, cytochrome c to oxygen | 5 | 1.42🞨10^-8^ | 6.60🞨10^-8^ |
| GO:0051092 | positive regulation of NF-kappaB transcription factor activity | 16 | 2.45🞨10^-8^ | 1.09🞨10^-7^ |
| GO:0042088 | T-helper 1 type immune response | 10 | 2.56🞨10^-8^ | 1.13🞨10^-7^ |
| GO:0034375 | high-density lipoprotein particle remodeling | 7 | 2.94🞨10^-8^ | 1.28🞨10^-7^ |
| GO:0051384 | response to glucocorticoid stimulus | 18 | 3.84🞨10^-8^ | 1.64🞨10^-7^ |
| GO:0002576 | platelet degranulation | 14 | 4.36🞨10^-8^ | 1.84🞨10^-7^ |
| GO:0032722 | positive regulation of chemokine production | 9 | 4.42🞨10^-8^ | 1.86🞨10^-7^ |
| GO:0044130 | negative regulation of growth of symbiont in host | 7 | 5.39🞨10^-8^ | 2.24🞨10^-7^ |
| GO:0006516 | glycoprotein catabolic process | 8 | 6.16🞨10^-8^ | 2.54🞨10^-7^ |
| GO:0001974 | blood vessel remodeling | 10 | 7.74🞨10^-8^ | 3.14🞨10^-7^ |
| GO:0010887 | negative regulation of cholesterol storage | 5 | 8.32🞨10^-8^ | 3.36🞨10^-7^ |
| GO:0032725 | positive regulation of granulocyte macrophage colony-stimulating factor production | 5 | 8.32🞨10^-8^ | 3.36🞨10^-7^ |
| GO:0034447 | very-low-density lipoprotein particle clearance | 5 | 8.32🞨10^-8^ | 3.36🞨10^-7^ |
| GO:0035094 | response to nicotine | 9 | 8.47🞨10^-8^ | 3.39🞨10^-7^ |
| GO:0031293 | membrane protein intracellular domain proteolysis | 7 | 9.36🞨10^-8^ | 3.68🞨10^-7^ |
| GO:0010332 | response to gamma radiation | 10 | 9.99🞨10^-8^ | 3.91🞨10^-7^ |
| GO:0045944 | positive regulation of transcription from RNA polymerase II promoter | 44 | 1.01🞨10^-7^ | 3.94🞨10^-7^ |
| GO:0032729 | positive regulation of interferon-gamma production | 9 | 1.15🞨10^-7^ | 4.42🞨10^-7^ |
| GO:0009612 | response to mechanical stimulus | 17 | 1.25🞨10^-7^ | 4.78🞨10^-7^ |
| GO:0034311 | diol metabolic process | 10 | 1.28🞨10^-7^ | 4.85🞨10^-7^ |
| GO:0050777 | negative regulation of immune response | 11 | 1.37🞨10^-7^ | 5.16🞨10^-7^ |
| GO:0010875 | positive regulation of cholesterol efflux | 6 | 1.56🞨10^-7^ | 5.82🞨10^-7^ |
| GO:0050435 | beta-amyloid metabolic process | 6 | 1.56🞨10^-7^ | 5.82🞨10^-7^ |
| GO:0050715 | positive regulation of cytokine secretion | 11 | 2.04🞨10^-7^ | 7.41🞨10^-7^ |
| GO:0042417 | dopamine metabolic process | 8 | 2.74🞨10^-7^ | 9.71🞨10^-7^ |
| GO:0030730 | sequestering of triglyceride | 6 | 3.05🞨10^-7^ | 1.07🞨10^-6^ |
| GO:0032355 | response to estradiol stimulus | 14 | 3.72🞨10^-7^ | 1.29🞨10^-6^ |
| GO:0048662 | negative regulation of smooth muscle cell proliferation | 8 | 3.80🞨10^-7^ | 1.31🞨10^-6^ |
| GO:0031663 | lipopolysaccharide-mediated signaling pathway | 9 | 4.53🞨10^-7^ | 1.54🞨10^-6^ |
| GO:0010745 | negative regulation of macrophage derived foam cell differentiation | 6 | 5.53🞨10^-7^ | 1.84🞨10^-6^ |
| GO:0055094 | response to lipoprotein particle stimulus | 6 | 5.53🞨10^-7^ | 1.84🞨10^-6^ |
| GO:0007220 | Notch receptor processing | 7 | 5.77🞨10^-7^ | 1.91🞨10^-6^ |
| GO:0032700 | negative regulation of interleukin-17 production | 5 | 7.42🞨10^-7^ | 2.40🞨10^-6^ |
| GO:0042136 | neurotransmitter biosynthetic process | 6 | 9.46🞨10^-7^ | 2.98🞨10^-6^ |
| GO:0043524 | negative regulation of neuron apoptotic process | 13 | 9.74🞨10^-7^ | 3.06🞨10^-6^ |
| GO:0032735 | positive regulation of interleukin-12 production | 7 | 1.21🞨10^-6^ | 3.73🞨10^-6^ |
| GO:0046470 | phosphatidylcholine metabolic process | 11 | 1.40🞨10^-6^ | 4.25🞨10^-6^ |
| GO:0034377 | plasma lipoprotein particle assembly | 6 | 1.54🞨10^-6^ | 4.61🞨10^-6^ |
| GO:0010872 | regulation of cholesterol esterification | 5 | 1.63🞨10^-6^ | 4.80🞨10^-6^ |
| GO:0032367 | intracellular cholesterol transport | 5 | 1.63🞨10^-6^ | 4.80🞨10^-6^ |
| GO:0009409 | response to cold | 8 | 2.06🞨10^-6^ | 5.99🞨10^-6^ |
| GO:0009408 | response to heat | 11 | 2.21🞨10^-6^ | 6.39🞨10^-6^ |
| GO:0045766 | positive regulation of angiogenesis | 12 | 2.24🞨10^-6^ | 6.47🞨10^-6^ |
| GO:0044704 | single-organism reproductive behavior | 11 | 2.56🞨10^-6^ | 7.32🞨10^-6^ |
| GO:0034114 | regulation of heterotypic cell-cell adhesion | 5 | 3.19🞨10^-6^ | 8.86🞨10^-6^ |
| GO:0034391 | regulation of smooth muscle cell apoptotic process | 5 | 3.19🞨10^-6^ | 8.86🞨10^-6^ |
| GO:0070587 | regulation of cell-cell adhesion involved in gastrulation | 5 | 3.19🞨10^-6^ | 8.86🞨10^-6^ |
| GO:0031331 | positive regulation of cellular catabolic process | 13 | 3.63🞨10^-6^ | 9.91🞨10^-6^ |
| GO:0061001 | regulation of dendritic spine morphogenesis | 6 | 3.64🞨10^-6^ | 9.91🞨10^-6^ |
| GO:0061082 | myeloid leukocyte cytokine production | 6 | 3.64🞨10^-6^ | 9.91🞨10^-6^ |
| GO:0042108 | positive regulation of cytokine biosynthetic process | 10 | 4.25🞨10^-6^ | 1.14🞨10^-5^ |
| GO:0040014 | regulation of multicellular organism growth | 11 | 4.50🞨10^-6^ | 1.20🞨10^-5^ |
| GO:0060333 | interferon-gamma-mediated signaling pathway | 11 | 5.14🞨10^-6^ | 1.36🞨10^-5^ |
| GO:0001660 | fever generation | 5 | 5.72🞨10^-6^ | 1.49🞨10^-5^ |
| GO:0034374 | low-density lipoprotein particle remodeling | 5 | 5.72🞨10^-6^ | 1.49🞨10^-5^ |
| GO:0002690 | positive regulation of leukocyte chemotaxis | 9 | 6.68🞨10^-6^ | 1.72🞨10^-5^ |
| GO:0033280 | response to vitamin D | 7 | 7.31🞨10^-6^ | 1.87🞨10^-5^ |
| GO:0044743 | intracellular protein transmembrane import | 20 | 8.13🞨10^-6^ | 2.07🞨10^-5^ |
| GO:0051971 | positive regulation of transmission of nerve impulse | 9 | 9.35🞨10^-6^ | 2.34🞨10^-5^ |
| GO:0045540 | regulation of cholesterol biosynthetic process | 5 | 9.59🞨10^-6^ | 2.39🞨10^-5^ |
| GO:0051769 | regulation of nitric-oxide synthase biosynthetic process | 5 | 9.59🞨10^-6^ | 2.39🞨10^-5^ |
| GO:0006805 | xenobiotic metabolic process | 15 | 9.90🞨10^-6^ | 2.46🞨10^-5^ |
| GO:0048661 | positive regulation of smooth muscle cell proliferation | 8 | 1.17🞨10^-5^ | 2.86🞨10^-5^ |
| GO:0051602 | response to electrical stimulus | 7 | 1.20🞨10^-5^ | 2.92🞨10^-5^ |
| GO:0051702 | interaction with symbiont | 7 | 1.20🞨10^-5^ | 2.92🞨10^-5^ |
| GO:0060740 | prostate gland epithelium morphogenesis | 7 | 1.20🞨10^-5^ | 2.92🞨10^-5^ |
| GO:0071248 | cellular response to metal ion | 11 | 1.24🞨10^-5^ | 3.01🞨10^-5^ |
| GO:0042346 | positive regulation of NF-kappaB import into nucleus | 6 | 1.46🞨10^-5^ | 3.48🞨10^-5^ |
| GO:2000401 | regulation of lymphocyte migration | 6 | 1.46🞨10^-5^ | 3.48🞨10^-5^ |
| GO:0010893 | positive regulation of steroid biosynthetic process | 5 | 1.52🞨10^-5^ | 3.60🞨10^-5^ |
| GO:0045073 | regulation of chemokine biosynthetic process | 5 | 1.52🞨10^-5^ | 3.60🞨10^-5^ |
| GO:0006120 | mitochondrial electron transport, NADH to ubiquinone | 8 | 1.70🞨10^-5^ | 3.98🞨10^-5^ |
| GO:0033138 | positive regulation of peptidyl-serine phosphorylation | 8 | 1.70🞨10^-5^ | 3.98🞨10^-5^ |
| GO:0002889 | regulation of immunoglobulin mediated immune response | 7 | 1.89🞨10^-5^ | 4.40🞨10^-5^ |
| GO:0002675 | positive regulation of acute inflammatory response | 6 | 1.96🞨10^-5^ | 4.52🞨10^-5^ |
| GO:0007271 | synaptic transmission, cholinergic | 6 | 1.96🞨10^-5^ | 4.52🞨10^-5^ |
| GO:0045907 | positive regulation of vasoconstriction | 6 | 1.96🞨10^-5^ | 4.52🞨10^-5^ |
| GO:0070328 | triglyceride homeostasis | 6 | 1.96🞨10^-5^ | 4.52🞨10^-5^ |
| GO:0001916 | positive regulation of T cell mediated cytotoxicity | 5 | 2.32🞨10^-5^ | 5.23🞨10^-5^ |
| GO:0032225 | regulation of synaptic transmission, dopaminergic | 5 | 2.32🞨10^-5^ | 5.23🞨10^-5^ |
| GO:0045723 | positive regulation of fatty acid biosynthetic process | 5 | 2.32🞨10^-5^ | 5.23🞨10^-5^ |
| GO:2000178 | negative regulation of neural precursor cell proliferation | 5 | 2.32🞨10^-5^ | 5.23🞨10^-5^ |
| GO:0006940 | regulation of smooth muscle contraction | 8 | 2.41🞨10^-5^ | 5.40🞨10^-5^ |
| GO:0032731 | positive regulation of interleukin-1 beta production | 6 | 2.59🞨10^-5^ | 5.75🞨10^-5^ |
| GO:0042596 | fear response | 6 | 2.59🞨10^-5^ | 5.75🞨10^-5^ |
| GO:0002637 | regulation of immunoglobulin production | 7 | 2.89🞨10^-5^ | 6.34🞨10^-5^ |
| GO:0012502 | induction of programmed cell death | 17 | 3.17🞨10^-5^ | 6.91🞨10^-5^ |
| GO:0002286 | T cell activation involved in immune response | 8 | 3.35🞨10^-5^ | 7.23🞨10^-5^ |
| GO:0046777 | protein autophosphorylation | 16 | 3.35🞨10^-5^ | 7.23🞨10^-5^ |
| GO:0048806 | genitalia development | 8 | 3.35🞨10^-5^ | 7.23🞨10^-5^ |
| GO:0019430 | removal of superoxide radicals | 5 | 3.40🞨10^-5^ | 7.31🞨10^-5^ |
| GO:0019886 | antigen processing and presentation of exogenous peptide antigen via MHC class II | 11 | 3.74🞨10^-5^ | 8.01🞨10^-5^ |
| GO:0048011 | nerve growth factor receptor signaling pathway | 18 | 4.19🞨10^-5^ | 8.92🞨10^-5^ |
| GO:0050830 | defense response to Gram-positive bacterium | 7 | 4.28🞨10^-5^ | 9.07🞨10^-5^ |
| GO:0014073 | response to tropane | 6 | 4.33🞨10^-5^ | 9.14🞨10^-5^ |
| GO:0032689 | negative regulation of interferon-gamma production | 6 | 4.33🞨10^-5^ | 9.14🞨10^-5^ |
| GO:0042220 | response to cocaine | 6 | 4.33🞨10^-5^ | 9.14🞨10^-5^ |
| GO:0050806 | positive regulation of synaptic transmission | 8 | 4.58🞨10^-5^ | 9.62🞨10^-5^ |
| GO:0051899 | membrane depolarization | 10 | 4.60🞨10^-5^ | 9.65🞨10^-5^ |
| GO:0042523 | positive regulation of tyrosine phosphorylation of Stat5 protein | 5 | 4.84🞨10^-5^ | 0.0001 |
| GO:0051926 | negative regulation of calcium ion transport | 5 | 4.84🞨10^-5^ | 0.0001 |
| GO:0090208 | positive regulation of triglyceride metabolic process | 5 | 4.84🞨10^-5^ | 0.0001 |
| GO:0043407 | negative regulation of MAP kinase activity | 9 | 5.26🞨10^-5^ | 0.0001 |
| GO:0009595 | detection of biotic stimulus | 6 | 5.51🞨10^-5^ | 0.0001 |
| GO:0010259 | multicellular organismal aging | 6 | 5.51🞨10^-5^ | 0.0001 |
| GO:0007422 | peripheral nervous system development | 10 | 5.73🞨10^-5^ | 0.0001 |
| GO:0050709 | negative regulation of protein secretion | 7 | 6.17🞨10^-5^ | 0.0001 |
| GO:0030449 | regulation of complement activation | 5 | 6.70🞨10^-5^ | 0.0001 |
| GO:0032733 | positive regulation of interleukin-10 production | 5 | 6.70🞨10^-5^ | 0.0001 |
| GO:0032770 | positive regulation of monooxygenase activity | 5 | 6.70🞨10^-5^ | 0.0001 |
| GO:0050829 | defense response to Gram-negative bacterium | 5 | 6.70🞨10^-5^ | 0.0001 |
| GO:0001960 | negative regulation of cytokine-mediated signaling pathway | 6 | 6.92🞨10^-5^ | 0.0001 |
| GO:0003044 | regulation of systemic arterial blood pressure mediated by a chemical signal | 7 | 7.35🞨10^-5^ | 0.0001 |
| GO:0002698 | negative regulation of immune effector process | 8 | 8.19🞨10^-5^ | 0.0002 |
| GO:0032570 | response to progesterone stimulus | 6 | 8.61🞨10^-5^ | 0.0002 |
| GO:0042092 | type 2 immune response | 6 | 8.61🞨10^-5^ | 0.0002 |
| GO:0000060 | protein import into nucleus, translocation | 7 | 8.70🞨10^-5^ | 0.0002 |
| GO:0014823 | response to activity | 7 | 8.70🞨10^-5^ | 0.0002 |
| GO:0030324 | lung development | 14 | 8.76🞨10^-5^ | 0.0002 |
| GO:0045671 | negative regulation of osteoclast differentiation | 5 | 9.07🞨10^-5^ | 0.0002 |
| GO:0045909 | positive regulation of vasodilation | 5 | 9.07🞨10^-5^ | 0.0002 |
| GO:0072678 | T cell migration | 5 | 9.07🞨10^-5^ | 0.0002 |
| GO:0021766 | hippocampus development | 8 | 9.39🞨10^-5^ | 0.0002 |
| GO:0022600 | digestive system process | 8 | 9.39🞨10^-5^ | 0.0002 |
| GO:0002544 | chronic inflammatory response | 5 | 0.0001 | 0.0002 |
| GO:0002720 | positive regulation of cytokine production involved in immune response | 5 | 0.0001 | 0.0002 |
| GO:0006919 | activation of cysteine-type endopeptidase activity involved in apoptotic process | 10 | 0.0001 | 0.0002 |
| GO:0007190 | activation of adenylate cyclase activity | 6 | 0.0001 | 0.0002 |
| GO:0007569 | cell aging | 9 | 0.0001 | 0.0002 |
| GO:0010575 | positive regulation vascular endothelial growth factor production | 5 | 0.0001 | 0.0002 |
| GO:0032846 | positive regulation of homeostatic process | 9 | 0.0001 | 0.0002 |
| GO:0042104 | positive regulation of activated T cell proliferation | 5 | 0.0001 | 0.0002 |
| GO:0043525 | positive regulation of neuron apoptotic process | 7 | 0.0001 | 0.0002 |
| GO:0045833 | negative regulation of lipid metabolic process | 8 | 0.0001 | 0.0002 |
| GO:0050706 | regulation of interleukin-1 beta secretion | 5 | 0.0001 | 0.0002 |
| GO:0050766 | positive regulation of phagocytosis | 6 | 0.0001 | 0.0002 |
| GO:0050996 | positive regulation of lipid catabolic process | 5 | 0.0001 | 0.0002 |
| GO:0051930 | regulation of sensory perception of pain | 5 | 0.0001 | 0.0002 |
| GO:0070374 | positive regulation of ERK1 and ERK2 cascade | 10 | 0.0001 | 0.0002 |
| GO:0002861 | regulation of inflammatory response to antigenic stimulus | 5 | 0.0002 | 0.0004 |
| GO:0008542 | visual learning | 6 | 0.0002 | 0.0004 |
| GO:0014047 | glutamate secretion | 6 | 0.0002 | 0.0004 |
| GO:0021795 | cerebral cortex cell migration | 6 | 0.0002 | 0.0004 |
| GO:0030279 | negative regulation of ossification | 6 | 0.0002 | 0.0004 |
| GO:0032757 | positive regulation of interleukin-8 production | 5 | 0.0002 | 0.0004 |
| GO:0046503 | glycerolipid catabolic process | 6 | 0.0002 | 0.0004 |
| GO:0048247 | lymphocyte chemotaxis | 5 | 0.0002 | 0.0004 |
| GO:0051279 | regulation of release of sequestered calcium ion into cytosol | 6 | 0.0002 | 0.0004 |
| GO:0055012 | ventricular cardiac muscle cell differentiation | 5 | 0.0002 | 0.0004 |
| GO:0070301 | cellular response to hydrogen peroxide | 7 | 0.0002 | 0.0004 |
| GO:0002886 | regulation of myeloid leukocyte mediated immunity | 5 | 0.0003 | 0.0005 |
| GO:0008344 | adult locomotory behavior | 8 | 0.0003 | 0.0005 |
| GO:0010039 | response to iron ion | 5 | 0.0003 | 0.0005 |
| GO:0015872 | dopamine transport | 5 | 0.0003 | 0.0005 |
| GO:0031334 | positive regulation of protein complex assembly | 10 | 0.0003 | 0.0005 |
| GO:0032091 | negative regulation of protein binding | 6 | 0.0003 | 0.0005 |
| GO:0032459 | regulation of protein oligomerization | 5 | 0.0003 | 0.0005 |
| GO:0034105 | positive regulation of tissue remodeling | 5 | 0.0003 | 0.0005 |
| GO:0043300 | regulation of leukocyte degranulation | 5 | 0.0003 | 0.0005 |
| GO:0045776 | negative regulation of blood pressure | 6 | 0.0003 | 0.0005 |
| GO:0050852 | T cell receptor signaling pathway | 10 | 0.0003 | 0.0005 |
| GO:0051953 | negative regulation of amine transport | 5 | 0.0003 | 0.0005 |
| GO:0051955 | regulation of amino acid transport | 5 | 0.0003 | 0.0005 |
| GO:0060444 | branching involved in mammary gland duct morphogenesis | 5 | 0.0003 | 0.0005 |
| GO:0060688 | regulation of morphogenesis of a branching structure | 7 | 0.0003 | 0.0005 |
| GO:0001954 | positive regulation of cell-matrix adhesion | 5 | 0.0004 | 0.0007 |
| GO:0002275 | myeloid cell activation involved in immune response | 7 | 0.0004 | 0.0007 |
| GO:0010469 | regulation of receptor activity | 8 | 0.0004 | 0.0007 |
| GO:0030888 | regulation of B cell proliferation | 7 | 0.0004 | 0.0007 |
| GO:0042517 | positive regulation of tyrosine phosphorylation of Stat3 protein | 5 | 0.0004 | 0.0007 |
| GO:0042638 | exogen | 14 | 0.0004 | 0.0007 |
| GO:0045806 | negative regulation of endocytosis | 5 | 0.0004 | 0.0007 |
| GO:0048265 | response to pain | 5 | 0.0004 | 0.0007 |
| GO:0048741 | skeletal muscle fiber development | 9 | 0.0004 | 0.0007 |
| GO:0060079 | regulation of excitatory postsynaptic membrane potential | 6 | 0.0004 | 0.0007 |
| GO:0002532 | production of molecular mediator involved in inflammatory response | 5 | 0.0005 | 0.0008 |
| GO:0007595 | lactation | 6 | 0.0005 | 0.0008 |
| GO:0009267 | cellular response to starvation | 9 | 0.0005 | 0.0008 |
| GO:0010712 | regulation of collagen metabolic process | 5 | 0.0005 | 0.0008 |
| GO:0019048 | virus-host interaction | 22 | 0.0005 | 0.0008 |
| GO:0044253 | positive regulation of multicellular organismal metabolic process | 5 | 0.0005 | 0.0008 |
| GO:0001975 | response to amphetamine | 5 | 0.0006 | 0.001 |
| GO:0007566 | embryo implantation | 6 | 0.0006 | 0.001 |
| GO:0010676 | positive regulation of cellular carbohydrate metabolic process | 6 | 0.0006 | 0.001 |
| GO:0034764 | positive regulation of transmembrane transport | 8 | 0.0006 | 0.001 |
| GO:0045912 | negative regulation of carbohydrate metabolic process | 5 | 0.0006 | 0.001 |
| GO:0050869 | negative regulation of B cell activation | 5 | 0.0006 | 0.001 |
| GO:0000122 | negative regulation of transcription from RNA polymerase II promoter | 26 | 0.0007 | 0.0011 |
| GO:0002548 | monocyte chemotaxis | 5 | 0.0007 | 0.0011 |
| GO:0031100 | organ regeneration | 7 | 0.0007 | 0.0011 |
| GO:0031623 | receptor internalization | 7 | 0.0007 | 0.0011 |
| GO:0050871 | positive regulation of B cell activation | 7 | 0.0007 | 0.0011 |
| GO:1900117 | regulation of execution phase of apoptosis | 13 | 0.0007 | 0.0011 |
| GO:2000027 | regulation of organ morphogenesis | 12 | 0.0007 | 0.0011 |
| GO:0001541 | ovarian follicle development | 7 | 0.0008 | 0.0012 |
| GO:0003401 | axis elongation | 5 | 0.0008 | 0.0012 |
| GO:0032309 | icosanoid secretion | 5 | 0.0008 | 0.0012 |
| GO:0048872 | homeostasis of number of cells | 13 | 0.0008 | 0.0012 |
| GO:0050873 | brown fat cell differentiation | 5 | 0.0008 | 0.0012 |
| GO:0051004 | regulation of lipoprotein lipase activity | 5 | 0.0008 | 0.0012 |
| GO:0015909 | long-chain fatty acid transport | 6 | 0.0009 | 0.0014 |
| GO:0030183 | B cell differentiation | 8 | 0.0009 | 0.0014 |
| GO:0042130 | negative regulation of T cell proliferation | 6 | 0.0009 | 0.0014 |
| GO:0071456 | cellular response to hypoxia | 8 | 0.0009 | 0.0014 |
| GO:0002381 | immunoglobulin production involved in immunoglobulin mediated immune response | 6 | 0.001 | 0.0015 |
| GO:0021587 | cerebellum morphogenesis | 5 | 0.001 | 0.0015 |
| GO:0031103 | axon regeneration | 5 | 0.001 | 0.0015 |
| GO:0033238 | regulation of cellular amine metabolic process | 8 | 0.001 | 0.0015 |
| GO:0043534 | blood vessel endothelial cell migration | 7 | 0.001 | 0.0015 |
| GO:0044243 | multicellular organismal catabolic process | 5 | 0.001 | 0.0015 |
| GO:0045453 | bone resorption | 6 | 0.001 | 0.0015 |
| GO:0051897 | positive regulation of protein kinase B signaling cascade | 7 | 0.001 | 0.0015 |
| GO:0030500 | regulation of bone mineralization | 7 | 0.0011 | 0.0016 |
| GO:0031396 | regulation of protein ubiquitination | 13 | 0.0011 | 0.0016 |
| GO:0043500 | muscle adaptation | 6 | 0.0011 | 0.0016 |
| GO:0046635 | positive regulation of alpha-beta T cell activation | 6 | 0.0011 | 0.0016 |
| GO:0009395 | phospholipid catabolic process | 5 | 0.0012 | 0.0018 |
| GO:0030431 | sleep | 5 | 0.0012 | 0.0018 |
| GO:0034644 | cellular response to UV | 5 | 0.0012 | 0.0018 |
| GO:0046928 | regulation of neurotransmitter secretion | 5 | 0.0012 | 0.0018 |
| GO:0050433 | regulation of catecholamine secretion | 5 | 0.0012 | 0.0018 |
| GO:0051099 | positive regulation of binding | 8 | 0.0012 | 0.0018 |
| GO:0030193 | regulation of blood coagulation | 7 | 0.0013 | 0.0019 |
| GO:0043266 | regulation of potassium ion transport | 5 | 0.0013 | 0.0019 |
| GO:0045599 | negative regulation of fat cell differentiation | 5 | 0.0013 | 0.0019 |
| GO:0051966 | regulation of synaptic transmission, glutamatergic | 5 | 0.0013 | 0.0019 |
| GO:0071347 | cellular response to interleukin-1 | 6 | 0.0013 | 0.0019 |
| GO:0001836 | release of cytochrome c from mitochondria | 6 | 0.0014 | 0.002 |
| GO:0043154 | negative regulation of cysteine-type endopeptidase activity involved in apoptotic process | 7 | 0.0014 | 0.002 |
| GO:0045732 | positive regulation of protein catabolic process | 8 | 0.0014 | 0.002 |
| GO:0061097 | regulation of protein tyrosine kinase activity | 6 | 0.0014 | 0.002 |
| GO:0009581 | detection of external stimulus | 9 | 0.0015 | 0.0022 |
| GO:0035023 | regulation of Rho protein signal transduction | 12 | 0.0016 | 0.0023 |
| GO:0048168 | regulation of neuronal synaptic plasticity | 6 | 0.0016 | 0.0023 |
| GO:0002064 | epithelial cell development | 8 | 0.0017 | 0.0024 |
| GO:0021675 | nerve development | 7 | 0.0017 | 0.0024 |
| GO:0007405 | neuroblast proliferation | 6 | 0.0018 | 0.0026 |
| GO:0031058 | positive regulation of histone modification | 5 | 0.0018 | 0.0026 |
| GO:0032885 | regulation of polysaccharide biosynthetic process | 5 | 0.0018 | 0.0026 |
| GO:0055013 | cardiac muscle cell development | 5 | 0.0018 | 0.0026 |
| GO:0007411 | axon guidance | 20 | 0.0019 | 0.0027 |
| GO:0006749 | glutathione metabolic process | 6 | 0.002 | 0.0028 |
| GO:0071383 | cellular response to steroid hormone stimulus | 6 | 0.002 | 0.0028 |
| GO:0009620 | response to fungus | 5 | 0.0021 | 0.003 |
| GO:0033209 | tumor necrosis factor-mediated signaling pathway | 5 | 0.0021 | 0.003 |
| GO:0048008 | platelet-derived growth factor receptor signaling pathway | 5 | 0.0021 | 0.003 |
| GO:0030336 | negative regulation of cell migration | 10 | 0.0022 | 0.0031 |
| GO:0007631 | feeding behavior | 8 | 0.0024 | 0.0034 |
| GO:0046686 | response to cadmium ion | 5 | 0.0024 | 0.0034 |
| GO:0050848 | regulation of calcium-mediated signaling | 5 | 0.0024 | 0.0034 |
| GO:0050999 | regulation of nitric-oxide synthase activity | 5 | 0.0024 | 0.0034 |
| GO:0031330 | negative regulation of cellular catabolic process | 6 | 0.0027 | 0.0038 |
| GO:0035176 | social behavior | 5 | 0.0027 | 0.0038 |
| GO:0042743 | hydrogen peroxide metabolic process | 5 | 0.0027 | 0.0038 |
| GO:0045840 | positive regulation of mitosis | 5 | 0.0027 | 0.0038 |
| GO:0006367 | transcription initiation from RNA polymerase II promoter | 13 | 0.0028 | 0.0039 |
| GO:0017085 | response to insecticide | 9 | 0.0028 | 0.0039 |
| GO:0031295 | T cell costimulation | 7 | 0.003 | 0.0042 |
| GO:0046164 | alcohol catabolic process | 6 | 0.003 | 0.0042 |
| GO:0048565 | digestive tract development | 9 | 0.003 | 0.0042 |
| GO:0045165 | cell fate commitment | 14 | 0.0031 | 0.0043 |
| GO:2000241 | regulation of reproductive process | 13 | 0.0032 | 0.0044 |
| GO:0014065 | phosphatidylinositol 3-kinase cascade | 7 | 0.0033 | 0.0046 |
| GO:0018107 | peptidyl-threonine phosphorylation | 6 | 0.0033 | 0.0046 |
| GO:0007431 | salivary gland development | 5 | 0.0034 | 0.0047 |
| GO:0051592 | response to calcium ion | 8 | 0.0034 | 0.0047 |
| GO:0071230 | cellular response to amino acid stimulus | 5 | 0.0034 | 0.0047 |
| GO:0051591 | response to cAMP | 7 | 0.0035 | 0.0048 |
| GO:0008016 | regulation of heart contraction | 9 | 0.0036 | 0.0049 |
| GO:0035914 | skeletal muscle cell differentiation | 6 | 0.0036 | 0.0049 |
| GO:0045778 | positive regulation of ossification | 5 | 0.0038 | 0.0052 |
| GO:0046677 | response to antibiotic | 5 | 0.0038 | 0.0052 |
| GO:0007219 | Notch signaling pathway | 10 | 0.0041 | 0.0056 |
| GO:0006476 | protein deacetylation | 5 | 0.0043 | 0.0058 |
| GO:0007041 | lysosomal transport | 5 | 0.0043 | 0.0058 |
| GO:0032663 | regulation of interleukin-2 production | 5 | 0.0043 | 0.0058 |
| GO:0043467 | regulation of generation of precursor metabolites and energy | 6 | 0.0043 | 0.0058 |
| GO:0048645 | organ formation | 6 | 0.0043 | 0.0058 |
| GO:0051928 | positive regulation of calcium ion transport | 6 | 0.0047 | 0.0063 |
| GO:0006984 | ER-nucleus signaling pathway | 8 | 0.0048 | 0.0064 |
| GO:0046324 | regulation of glucose import | 5 | 0.0048 | 0.0064 |
| GO:0051145 | smooth muscle cell differentiation | 5 | 0.0048 | 0.0064 |
| GO:0071333 | cellular response to glucose stimulus | 5 | 0.0048 | 0.0064 |
| GO:0006027 | glycosaminoglycan catabolic process | 6 | 0.0051 | 0.0068 |
| GO:0010906 | regulation of glucose metabolic process | 7 | 0.0051 | 0.0068 |
| GO:0010976 | positive regulation of neuron projection development | 6 | 0.0051 | 0.0068 |
| GO:0043507 | positive regulation of JUN kinase activity | 6 | 0.0051 | 0.0068 |
| GO:0006611 | protein export from nucleus | 5 | 0.0053 | 0.007 |
| GO:0048641 | regulation of skeletal muscle tissue development | 6 | 0.0055 | 0.0073 |
| GO:0008286 | insulin receptor signaling pathway | 11 | 0.0056 | 0.0074 |
| GO:0002474 | antigen processing and presentation of peptide antigen via MHC class I | 8 | 0.0057 | 0.0075 |
| GO:0045445 | myoblast differentiation | 5 | 0.0059 | 0.0078 |
| GO:0050680 | negative regulation of epithelial cell proliferation | 7 | 0.0059 | 0.0078 |
| GO:0071418 | cellular response to amine stimulus | 5 | 0.0059 | 0.0078 |
| GO:0001764 | neuron migration | 8 | 0.0061 | 0.008 |
| GO:0008584 | male gonad development | 8 | 0.0064 | 0.0084 |
| GO:0009060 | aerobic respiration | 5 | 0.0065 | 0.0085 |
| GO:0048010 | vascular endothelial growth factor receptor signaling pathway | 5 | 0.0065 | 0.0085 |
| GO:0048145 | regulation of fibroblast proliferation | 6 | 0.0065 | 0.0085 |
| GO:0050770 | regulation of axonogenesis | 8 | 0.0068 | 0.0089 |
| GO:0000096 | sulfur amino acid metabolic process | 5 | 0.0071 | 0.0092 |
| GO:0033143 | regulation of intracellular steroid hormone receptor signaling pathway | 5 | 0.0071 | 0.0092 |
| GO:0042246 | tissue regeneration | 5 | 0.0071 | 0.0092 |
| GO:0042490 | mechanoreceptor differentiation | 5 | 0.0071 | 0.0092 |
| GO:0048259 | regulation of receptor-mediated endocytosis | 5 | 0.0071 | 0.0092 |
| GO:1901605 | alpha-amino acid metabolic process | 11 | 0.0075 | 0.0097 |
| GO:0045740 | positive regulation of DNA replication | 5 | 0.0078 | 0.0101 |
| GO:0042058 | regulation of epidermal growth factor receptor signaling pathway | 6 | 0.0082 | 0.0106 |
| GO:0046148 | pigment biosynthetic process | 5 | 0.0085 | 0.0109 |
| GO:0006941 | striated muscle contraction | 7 | 0.0087 | 0.0112 |
| GO:0003151 | outflow tract morphogenesis | 5 | 0.0093 | 0.0118 |
| GO:0007215 | glutamate receptor signaling pathway | 5 | 0.0093 | 0.0118 |
| GO:0035872 | nucleotide-binding domain, leucine rich repeat containing receptor signaling pathway | 5 | 0.0093 | 0.0118 |
| GO:0042308 | negative regulation of protein import into nucleus | 5 | 0.0093 | 0.0118 |
| GO:0045685 | regulation of glial cell differentiation | 5 | 0.0093 | 0.0118 |
| GO:0021536 | diencephalon development | 6 | 0.0094 | 0.0119 |
| GO:0043087 | regulation of GTPase activity | 7 | 0.0098 | 0.0124 |
| GO:0044344 | cellular response to fibroblast growth factor stimulus | 10 | 0.01 | 0.0126 |
| GO:0005977 | glycogen metabolic process | 6 | 0.0101 | 0.0127 |
| GO:0006879 | cellular iron ion homeostasis | 6 | 0.0101 | 0.0127 |
| GO:0001889 | liver development | 7 | 0.0104 | 0.0131 |
| GO:0006958 | complement activation, classical pathway | 5 | 0.0111 | 0.0139 |
| GO:0030593 | neutrophil chemotaxis | 5 | 0.0111 | 0.0139 |
| GO:0001822 | kidney development | 12 | 0.0112 | 0.014 |
| GO:0000186 | activation of MAPKK activity | 5 | 0.012 | 0.015 |
| GO:0021954 | central nervous system neuron development | 5 | 0.012 | 0.015 |
| GO:0050796 | regulation of insulin secretion | 9 | 0.0128 | 0.0159 |
| GO:0031018 | endocrine pancreas development | 5 | 0.013 | 0.0162 |
| GO:0001890 | placenta development | 8 | 0.0135 | 0.0167 |
| GO:0034754 | cellular hormone metabolic process | 7 | 0.0138 | 0.0171 |
| GO:0072657 | protein localization to membrane | 7 | 0.0138 | 0.0171 |
| GO:0048469 | cell maturation | 8 | 0.0142 | 0.0175 |
| GO:0045582 | positive regulation of T cell differentiation | 5 | 0.0151 | 0.0185 |
| GO:0055008 | cardiac muscle tissue morphogenesis | 5 | 0.0151 | 0.0185 |
| GO:0032526 | response to retinoic acid | 7 | 0.0162 | 0.0198 |
| GO:0051289 | protein homotetramerization | 5 | 0.0163 | 0.0199 |
| GO:0071887 | leukocyte apoptotic process | 5 | 0.0163 | 0.0199 |
| GO:0007416 | synapse assembly | 6 | 0.0169 | 0.0206 |
| GO:0019432 | triglyceride biosynthetic process | 5 | 0.0175 | 0.0213 |
| GO:0045665 | negative regulation of neuron differentiation | 5 | 0.0175 | 0.0213 |
| GO:0051149 | positive regulation of muscle cell differentiation | 5 | 0.0175 | 0.0213 |
| GO:0006970 | response to osmotic stress | 5 | 0.0188 | 0.0228 |
| GO:0034763 | negative regulation of transmembrane transport | 5 | 0.0188 | 0.0228 |
| GO:0044275 | cellular carbohydrate catabolic process | 5 | 0.0188 | 0.0228 |
| GO:0050886 | endocrine process | 5 | 0.0188 | 0.0228 |
| GO:0008360 | regulation of cell shape | 7 | 0.0207 | 0.0249 |
| GO:0009207 | purine ribonucleoside triphosphate catabolic process | 18 | 0.0207 | 0.0249 |
| GO:0002831 | regulation of response to biotic stimulus | 6 | 0.0224 | 0.0268 |
| GO:0008063 | Toll signaling pathway | 6 | 0.0224 | 0.0268 |
| GO:0001756 | somitogenesis | 5 | 0.0229 | 0.0273 |
| GO:0006094 | gluconeogenesis | 5 | 0.0229 | 0.0273 |
| GO:0042446 | hormone biosynthetic process | 5 | 0.0229 | 0.0273 |
| GO:0043648 | dicarboxylic acid metabolic process | 6 | 0.0236 | 0.0281 |
| GO:0050792 | regulation of viral reproduction | 7 | 0.0239 | 0.0285 |
| GO:0009154 | purine ribonucleotide catabolic process | 18 | 0.0242 | 0.0288 |
| GO:0009615 | response to virus | 13 | 0.025 | 0.0296 |
| GO:0046130 | purine ribonucleoside catabolic process | 18 | 0.0258 | 0.0305 |
| GO:0003208 | cardiac ventricle morphogenesis | 5 | 0.026 | 0.0307 |
| GO:0035050 | embryonic heart tube development | 5 | 0.026 | 0.0307 |
| GO:0045639 | positive regulation of myeloid cell differentiation | 5 | 0.026 | 0.0307 |
| GO:0097193 | intrinsic apoptotic signaling pathway | 7 | 0.0261 | 0.0308 |
| GO:0048515 | spermatid differentiation | 6 | 0.0263 | 0.031 |
| GO:0006096 | glycolysis | 5 | 0.0276 | 0.0323 |
| GO:0045727 | positive regulation of translation | 5 | 0.0276 | 0.0323 |
| GO:0033189 | response to vitamin A | 7 | 0.0286 | 0.0335 |
| GO:0030198 | extracellular matrix organization | 11 | 0.0289 | 0.0338 |
| GO:0006987 | activation of signaling protein activity involved in unfolded protein response | 5 | 0.0293 | 0.0341 |
| GO:0009206 | purine ribonucleoside triphosphate biosynthetic process | 5 | 0.0293 | 0.0341 |
| GO:0046620 | regulation of organ growth | 5 | 0.0293 | 0.0341 |
| GO:0051101 | regulation of DNA binding | 5 | 0.0293 | 0.0341 |
| GO:0051153 | regulation of striated muscle cell differentiation | 5 | 0.0293 | 0.0341 |
| GO:0001935 | endothelial cell proliferation | 6 | 0.0305 | 0.0354 |
| GO:0045744 | negative regulation of G-protein coupled receptor protein signaling pathway | 5 | 0.0311 | 0.036 |
| GO:0001508 | regulation of action potential | 8 | 0.0316 | 0.0365 |
| GO:0009791 | post-embryonic development | 6 | 0.032 | 0.037 |
| GO:0032479 | regulation of type I interferon production | 5 | 0.0329 | 0.0379 |
| GO:2001233 | regulation of apoptotic signaling pathway | 5 | 0.0329 | 0.0379 |
| GO:0034142 | toll-like receptor 4 signaling pathway | 6 | 0.0336 | 0.0387 |
| GO:0007188 | adenylate cyclase-modulating G-protein coupled receptor signaling pathway | 7 | 0.0338 | 0.0388 |
| GO:0001649 | osteoblast differentiation | 8 | 0.0341 | 0.0392 |
| GO:0034637 | cellular carbohydrate biosynthetic process | 5 | 0.0348 | 0.0399 |
| GO:0035666 | TRIF-dependent toll-like receptor signaling pathway | 5 | 0.0348 | 0.0399 |
| GO:0050821 | protein stabilization | 5 | 0.0348 | 0.0399 |
| GO:0060349 | bone morphogenesis | 5 | 0.0348 | 0.0399 |
| GO:0007292 | female gamete generation | 6 | 0.0352 | 0.0403 |
| GO:0007050 | cell cycle arrest | 17 | 0.0362 | 0.0414 |
| GO:0030308 | negative regulation of cell growth | 8 | 0.0366 | 0.0418 |
| GO:0043433 | negative regulation of sequence-specific DNA binding transcription factor activity | 7 | 0.0367 | 0.0419 |
| GO:0001570 | vasculogenesis | 5 | 0.0368 | 0.0419 |
| GO:0031333 | negative regulation of protein complex assembly | 5 | 0.0368 | 0.0419 |
| GO:0034130 | toll-like receptor 1 signaling pathway | 5 | 0.0368 | 0.0419 |
| GO:0048489 | synaptic vesicle transport | 5 | 0.0368 | 0.0419 |
| GO:0010921 | regulation of phosphatase activity | 6 | 0.0386 | 0.0438 |
| GO:0030307 | positive regulation of cell growth | 6 | 0.0386 | 0.0438 |
| GO:0032886 | regulation of microtubule-based process | 6 | 0.0386 | 0.0438 |
| GO:0006720 | isoprenoid metabolic process | 5 | 0.0388 | 0.044 |
| GO:0046039 | GTP metabolic process | 15 | 0.0405 | 0.0459 |
| GO:0007179 | transforming growth factor beta receptor signaling pathway | 9 | 0.0406 | 0.046 |
| GO:0003014 | renal system process | 5 | 0.0409 | 0.0462 |
| GO:0032412 | regulation of ion transmembrane transporter activity | 5 | 0.0409 | 0.0462 |
| GO:0048675 | axon extension | 5 | 0.0409 | 0.0462 |
| GO:0050679 | positive regulation of epithelial cell proliferation | 7 | 0.0413 | 0.0466 |
| GO:0090101 | negative regulation of transmembrane receptor protein serine/threonine kinase signaling pathway | 6 | 0.0422 | 0.0475 |
| GO:0016197 | endosomal transport | 8 | 0.0436 | 0.049 |
| GO:0043623 | cellular protein complex assembly | 14 | 0.0441 | 0.0496 |

^a^ Alzgset: Alzheimer’s disease related genes gene set

^b^ Only the leaf GO Biological Process terms containing 5 or more genes among Alzgset are shown.

^c^ Number of genes among Alzgset and also in the category

^d^ P-values were calculated by hypergeometric test

^e^ P_BH_-values were adjusted by Benjamini & Hochberg (BH) method
